# Thirteen dubious ways to detect conserved structural RNAs

**DOI:** 10.1002/iub.2694

**Published:** 2022-12-10

**Authors:** William Gao, Ann Yang, Elena Rivas

**Affiliations:** 1 Department of Genetics, University of Pennsylvania, Philadelphia, Pennsylvania, USA; 2 Department of Molecular and Cellular Biology, Harvard University, Cambridge, Massachusetts, USA

## Abstract

Covariation induced by compensatory base substitutions in RNA alignments is a great way to deduce conserved RNA structure, in principle. In practice, success depends on many factors, importantly the quality and depth of the alignment and the choice of covariation statistic. Measuring covariation between pairs of aligned positions is easy. However, using covariation to infer evolutionarily conserved RNA structure is complicated by other extraneous sources of covariation such as that resulting from homologous sequences having evolved from a common ancestor. In order to provide evidence of evolutionarily conserved RNA structure, a method to distinguish covariation due to sources other than RNA structure is necessary. Moreover, there are several sorts of artifactually generated covariation signals that can further confound the analysis. Additionally, some covariation signal is difficult to detect due to incomplete comparative data. Here, we investigate and critically discuss the practice of inferring conserved RNA structure by comparative sequence analysis. We provide new methods on how to approach and decide which of the numerous long non-coding RNAs (lncRNAs) have biologically relevant structures.

## 1 | IS IT A CONSERVED RNA STRUCTURE, OR NOT? …OR CAN WE EVEN TELL?

In this article, we discuss how to identify conserved RNA structure, that is, how to determine if a genomic region is under selective pressure to maintain some particular RNA secondary structure.

Identifying evolutionarily conserved structural RNAs requires the use of comparative data. A conserved RNA structure results in a pattern of compensatory base pair substitutions that tend to preserve the base pairs.^1^ As a quantitative measure of the amount of compensatory base pair substitutions, covariation has been used very successfully to infer the structure of many fundamental structural RNAs such as tRNAs,^2^ ribosomal RNA,^3,4^ group I introns,^5^ and ribozymes,^6^ to name a few. Numerous computational methods have been designed to determine RNA structures using covariation.^7–11^ Many others have taken advantage of covariation in related tasks such as RNA homology searches,^12–14^ RNA structural alignments,^15,16^ and RNA genefinding.^17–20^ However, identifying novel conserved RNA structures is *a question fundamentally different* from RNA structure prediction, RNA homology searches or structural alignment.

Unlike structure prediction where we assume *there is* a conserved structure, and unlike homology search or alignment where we are happy to use primary sequence conservation alone, asserting the presence of a conserved RNA structure requires knowing if the covariation we see is more than what we expect from other biological explanations. The question is not what are the base pairs in this structure, but instead, does this RNA have a conserved RNA structure to start with?

### Identifying conserved RNA structure is necessarily a computational analysis question.

Essentially, any RNA has structure; SHAPE and other chemical probing methods^21–25^ will still give a signal in random sequences, and will not be able to distinguish a random sequence from one under selective pressure to conserve one or several particular structures. The experiment one would like to do to demonstrate the importance of a particular RNA structure is compensatory mutation analysis. That is, to show that single mutations that disrupt either side of an inferred base pair disrupt function, but a double mutant that restores base pairing also restores the function. Evolution has already done that experiment on a massive scale. Computationally, we can analyze the sequences that have successfully mutated and maintained structure and function in vivo.

### But why care specifically about conserved RNA structure?

As stated above and as reasoned by Vicens & Kieft,^26^ RNA sequences of any kind, including mRNAs or even random ones, will produce some folding under biological conditions. It is also likely that for a given transcript many different structures coexist, based on the many different theoretical folding possibilities that present comparable thermodynamic stabilities. The ensemble of structures, likely transient and dynamic, may be related to an overall functional state of the RNA. For instance, mRNA global structuredness seems to increase RNA stability, which has implications for translation efficiency.^27^ However, when RNA is involved in some specific function, often it relies on conserved RNA structures involved in processes conserved across species. There are many well-known classes of functional conserved structural RNAs involved in fundamental cellular functions. An excellent compilation of conserved RNA sequence and structure can be found in the database of RNA families Rfam.^28^ We hypothesize that other RNAs with conserved structures remain to be discovered which are likely to lead us to specific and perhaps novel cellular mechanisms.

Identifying novel conserved RNA structures is really *two computational tasks*: (1) To determine whether the amount of observed pairwise covariation is more than we expect if there is not a conserved structure. For this, we need a null hypothesis to compare to.^29,30^ (2) To determine how much pairwise covariation we expect to see if there were a conserved structure, given how much sequence variation there is available in the alignment because sometimes, with highly conserved regions, there will not be enough variation to know if there is an evolutionarily conserved structure. The first problem is about statistical significance, the second is about statistical power.

**FIGURE 1:**
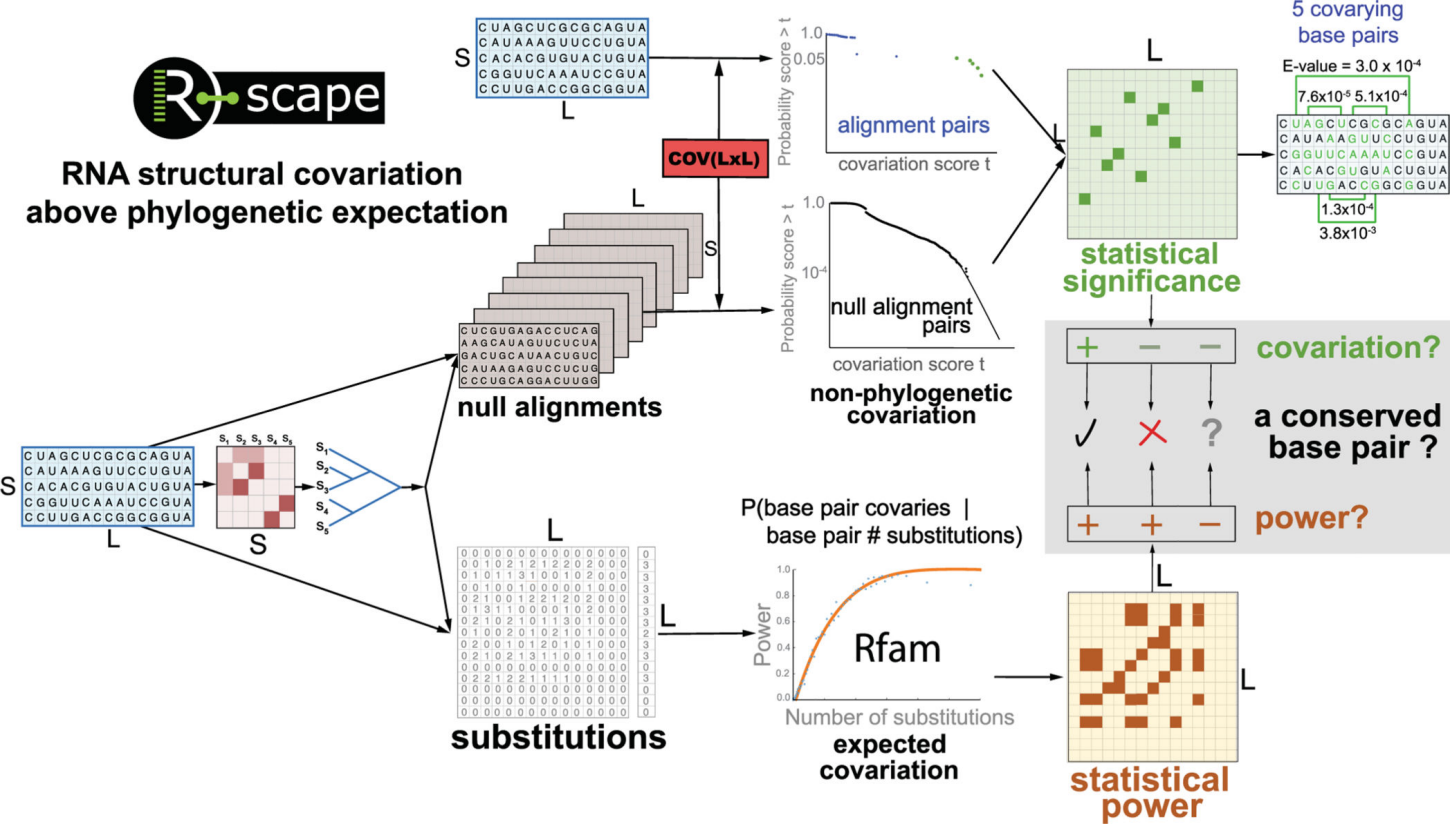
The comparative signals utilized by R-scape. We use a toy alignment of 5 sequences (S) and 15 nts in length (L) to show the principles behind R-scape. Given the alignment, a phylogenetic tree is produced. The alignment and the tree are used both to generate null alignments (Figure 3a), as well as to estimate the number of substitutions per position. The null alignments are used to generate a null distribution of covariation scores due strictly to the phylogenetic relationships amongst the sequences. The substitutions are used to estimate covariation power, that is, the probability of the pair being called significant if it were a base pair. R-scape uses both covariation and power to decide on whether the alignment supports a conserved structure, rejects the hypothesis of a conserved structure or cannot make any inference about whether there is a conserved RNA structure or not. For this toy alignment and using default setting, R-scape identifies five significantly covarying base pairs with E-values smaller than 0.05

We have developed a computational tool (R-scape) that addresses both questions,^31,32^ which is succinctly described in Figure 1. Here, we will use R-scape as a specific example, but the main considerations apply broadly.

For the *first task* (statistical significance), the main source of confounding pairwise covariation comes from the phylogenetic relatedness of the sequences. The covariation observed between pairs of columns in an input multiple sequence alignment is compared against that of related synthetic alignments generated under the null hypothesis, from which we can assign an E-value (that is, the expected number of null pairs of columns with a similar covariation score or bigger) for all pairs in the input alignment. We say that a base pair significantly covaries if it has an R-scape E-value <0.05, by default.

For the *second task* (statistical power), we use a source of true conserved structures such as Rfam.^28^ Statistical power is how much significant covariation to expect if there were a conserved structure given the sequence variation observed in the alignment. The power of a base pair is a probability between 0 and 1 that the base pair is expected to be detected as significantly covarying based on the variability that is present.

Using covariation and power we can distinguish between three different scenarios of RNA conservation: significant covariation indicates the presence of a conserved base pair. In the absence of covariation, power allows us to distinguish two other possible scenarios: lack of power together with lack of covariation means that the alignment is too conserved at the primary sequence level to give any information about whether there is a conserved structure or not. On the other hand, power in the absence of covariation is evidence against a conserved base pair.

Using covariation and power, we can re-shape the question of RNA structure inference. The combination of covariation and power lets us predict *negative pairs*. A negative pair has plenty of variation, enough that if the two aligned columns form a conserved base pair we would expect to see significant covariation, but there is not. Having negative pairs provides a novel constraint for RNA structure inference, and we recently described a method CaCoFold (Cascade variation/covariation Constrained Folding algorithm), that uses both positive and negative pair information to infer conserved structures. CaCoFold can propose any folding topology provided that it shows significant covariation. The amount of support from (significantly covarying) *positive pairs* provides a direct measure of the reliability of the proposed structure.^34^

Using R-scape (or conceptually related approaches) enables genome-wide screens for novel conserved RNA structures. In particular, it enables screens of long noncoding RNAs (lncRNAs) for conserved RNA structures. LncRNAs are mRNA-like transcripts of more than 200 nucleotidies characterized by the sole property that they do not appear to encode a protein. In humans alone, between ten and hundred thousand lncRNAs have been reported.^35–37^ These transcripts tend to be expressed at low levels,^38^ are rapidly degraded by the RNA exosome,^39,40^ and have on average longer transcriptional burst periods relative to mRNAs, resulting in larger cell-to-cell transcriptional variability.^41^

While only a handful of lncRNAs have been functionally characterized, it is often assumed that lncRNAs exert their function by means of RNA structure. While it is expected that any RNA may arrange in a compact folded state,^26^ it cannot be a priori assumed that any of these lncRNAs have any particular conserved structure that may serve a specific function.

Because of the large genome size and number of RNAs in an organism, especially vertebrates, an important issue affecting most previous methods’ ability to reliably detect structural RNAs is specificity, that is, false positive rate.^17–20^ The tally of structural lncRNAs has been affected by many difficulties that result from combining a signal difficult to characterize^42^ with the large size of vertebrate genomes and transcriptomes. Existing predictions of human structural RNAs are large and highly variable in numbers, with little overlap amongst different methods, and large numbers of estimated false positives.^43–46^

The general framework described in Figure 1 is an approach for interrogating transcripts or genomic regions and selecting the subset that is likely to have a conserved RNA structure. This method has controllable specificity as we showed in a recent analysis of novel ncRNAs in fungi, where we identified 21 new structural RNAs, 17 of which are novel, with an estimated specificity of 0.003 total false positives in a search over 134,000 alignments of conserved noncoding genomic regions, and 78% (62/80) sensitivity to detect known structural RNAs in *S. cerevisiae*.^47^

Controversy still remains over lncRNAs and how to decide which have biologically relevant structures. In our experience so far using R-scape at the genome scale, we have observed a variety of important sources of confounding covariation. Here, with our title inspired by a famous numerical analysis paper in the same spirit,* we aim to investigate the main practical issues in inferring the presence of an evolutionarily conserved RNA structure. Some issues have to do with the choice of covariation statistic and its compatibility with how the null hypothesis is chosen. Other issues have to do with the quality of the alignment from which the covariation signal is extracted. For each of these 13 issues, we provide examples, a way to identify similar artifacts, and a way forward.

## 2 | THIRTEEN ISSUES TO BEWARE OF

**FIGURE 2:**
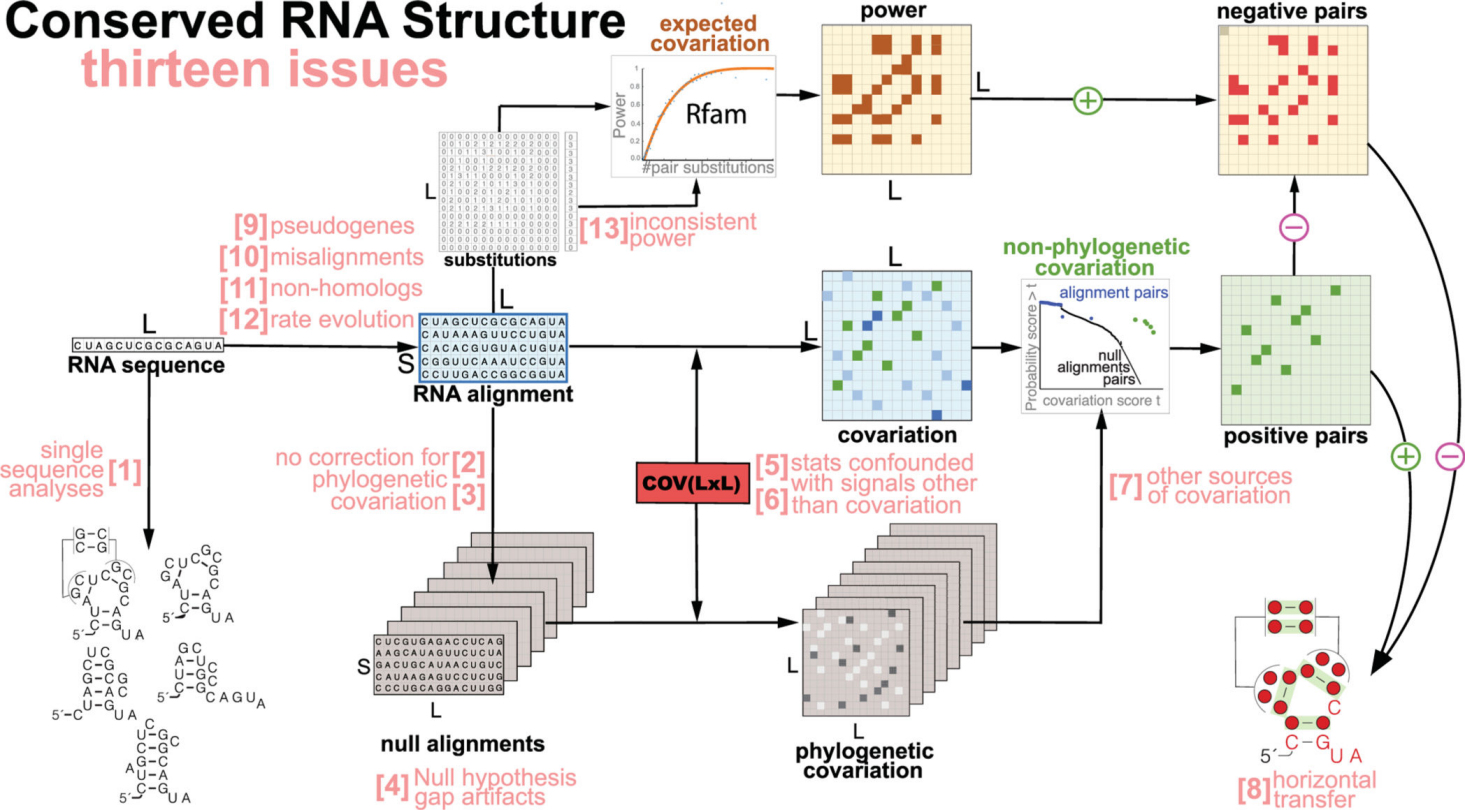
Thirteen issues to be aware of in the detection of conserved RNA structure. Given an RNA sequence of length L, such that the sequence is conserved forming an alignment of S homologs, several issues need to be considered in the analysis of whether the RNA has a conserved structure. **Issue [1]** refers to the need of using an alignment. **Two issues [2,3]** have to do with correcting for covariation not due to RNA structure. **Issue [4]** addresses the assumptions of the null hypothesis. **Two issues [5, 6]** have to do with using a good covariation measure compatible with the null hypothesis. **Issue [7]** refers to other sources of covariation that are not phylogeny or RNA structure. **Issue [8]** refers to when a conserved RNA structure may not indicate a functional RNA, for instance with the nuclear mitochondrial RNAs (NUMTs). Finally, **four issues [9, 10, 11, 12, 13]** have to do with the quality of the alignment and the evolutionary divergence of the comparative data

Here, we touch on different issues that could ultimately result in an erroneous call as to whether a given transcript conserves an RNA structure or not. Figure 2 provides an overview of the thirteen issues discussed below.

### 2.1 | [1] Beware of single-sequence analyses

Single-sequence analyses cannot inform about conserved RNA structure. An alignment is needed.

Single-sequence analyses typically use a computational RNA folding method^49–58^ constrained by chemical probing data obtained from high-throughput experiments either in vitro or in vivo.^23,59^ The computational method explores the most stable structures based on the sequence alone. The chemical probing provides a measure of base reactivities informative of the folded state of the RNA molecule. The combination of those two sources of information result in predicted structures for one single RNA sequence. By sampling from the distribution of possible structures, these single-sequence methods can report one or more as possible alternative structural conformations of the RNA molecule.^24,25,60^

Regardless of the quality of these single-sequence structural predictions, these methods do not provide any information regarding whether the structures are conserved or biologically relevant. Since all RNA molecules are bound to adopt some folded conformation,^26^ one should expect predictions of stable RNA structures in essentially any sequence. However, a *stable structure* is not equivalent to a *conserved structure*. Only comparative data can infer whether a structure is conserved through evolution.

As an example, the SARS-CoV-2 virus genome includes several known RNA structures, such as the 3′ and 5′ UTRs, the s2m RNA, a frameshift structure, and the packing signal. Recently, other structural elements have been predicted by the hundreds,^61^ but only a small fraction seems to be evolutionarily conserved.^62^ These evolutionarily conserved structures have a higher likelihood to be involved in some functional role.

### 2.2 | [2] Beware of phylogenetic covariation

Phylogenetic covariation needs to be taken into account because it creates a confounding covariation signal.

It has long been established that a conserved RNA structure induces covariation in an alignment. Different mathematical measures of covariation exist, such as mutual information (MI),^63^ G-test,^64^ and others.^65,66^ Using covariation information, the consensus structures of both subunits of ribosomal RNA were deduced, a huge success for two of the largest and most conserved structural RNA molecules known.^3,67,68^ However, the reciprocal is not true; covariation does not necessarily imply the presence of a conserved RNA structure. After all, most pairs of positions in an alignment have some amount of covariation. How much covariation is enough to decide whether there is evidence of a conserved base pair?

When we remember that the reason why the sequences are alignable is because that they share a common ancestor then we find another source of covariation not due to RNA structure. Independent substitutions can occur in two different sites in an ancestral sequence on one branch of the phylogeny. Because all descendants will tend to inherit both substitutions, the contrast between sequences in the clade with the substitutions versus other sequences without them appears to be a pairwise covariation signal when compared to simple measures that assume that sequences are independent.^30^ Phylogenetic covariation has to be taken into account in order to detect the evolutionary signal left behind by a conserved RNA structure.

**FIGURE 3:**
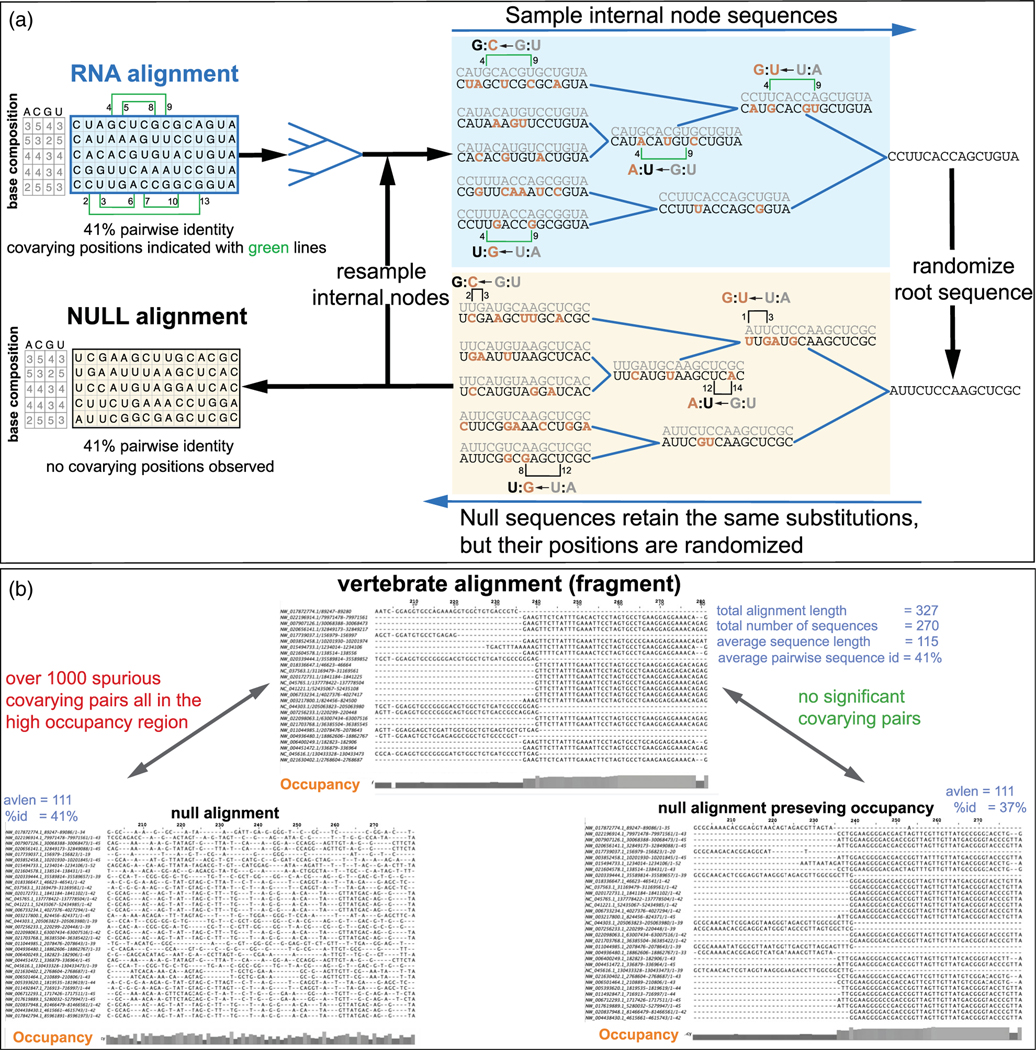
Accounting for phylogenetic covariation. (a) We show the process of generating alignments under the null hypothesis of covariation due solely to phylogeny using the toy alignment introduced in Figure 1. For a given input alignment (in blue, top left), we build a phylogenetic tree, for which different sets of internal node sequences can be sampled using Fitch’s algorithm.^33^ A particular sample of the three internal nodes for this toy alignment is given in blue (top right). At each node, we show the sampled sequence (in black) together with its ancestral sequence above (in gray), so that we can highlight the substitutions between the two (in brown). All null synthetic alignments follow the same phylogeny, but each null alignment is generated from a different sample of the input alignment’s internal nodes. An example null alignment (in yellow, bottom left) is generated introducing the same substitutions per branch than the given alignment, but the substitutions are applied at random positions in the corresponding null sequences (yellow, bottom right). As an example, we follow the base pair between positions 4:9 (one of the five significant base pairs indicated left with green lines). This 4:9 base pair includes five-base-pair-preserving substitutions occurring at four branches. The same five substitutions appear at the same four branches in the null alignment, but at positions different from 4:9 and selected at random from other available positions. Both alignments have similar average percentage identity (41%), and the null sequences have the same base composition than their equivalent ones in the input alignment. Per-column conservation however is not preserved. (b) An example alignment with a non-uniform gap distribution that results in biased occupancy, with the right side of the alignment having higher occupancy than its left side. A null alignment sampling method that does not preserve the gap distribution of the input alignment (bottom left) may score as significant residues in the right side of the alignment when we use a covariation measure such as G-test (R-scape’s default) that is sensitive to occupancy. The problem is resolved by using a null alignment sampling algorithm that preserves the alignment occupancy (bottom right).

We first introduced R-scape to empirically determine the distribution of covariation scores for null alignments such that covariation generated by the phylogeny (by independent substitutions, sometimes co-occurring in the same branch) are preserved while randomizing the site of those substitutions to destroy the sort of recurrent pairwise compensatory substitutions observed for structural covariation.^31^ The null alignments are constructed using the phylogenetic tree inferred for the input alignment. Each null alignment includes the same total number of substitutions per branch as the input alignment. However, for the null alignments, the sites of substitutions per branch are selected at random amongst all possible sites, thus destroying any existing site correlation in the input alignment but retaining the possible phylogenetic ones. For each input alignment, a collection of null alignments are synthesized and a distribution of expected covariation due to phylogeny is calculated. This distribution is used to estimate for each base pair in the input alignment an expected number (E-value) of just phylogenetically related pairs that could have similar covariation scores (Figure 3a).

### 2.3 | [3] Beware of what the null hypothesis destroys

The properties of the original alignment that are randomized away in the null alignments can appear as significant if measured–but may not be structural covariation.

Here, we show an example of an alignment property that may interfere with structural RNA significance.

The evolutionary signal of residue substitutions used in covariation analysis is almost always accompanied by insertions and deletions. Conserved RNA structures can have variable number of base pairs and even different numbers of helices. Those events introduce gaps in alignments. Covariation measures such as MI do not take gaps into account. MI is defined as the ratio of the joint ${\text{P}}_{ij}^{ab}$ to marginal residue probabilities for any pair of positions (i,j), with a,b={A,C,G,U},

$$\text{MI}(i,j)=\sum_{a,b}{\text{P}}_{ij}^{ab}\text{log}\frac{{\text{P}}_{ij}^{ab}}{{\text{P}}_{i}^{a}{\text{P}}_{j}^{b}}.$$

On the other hand, G-test, another measure of pairwise covariation, uses a similar expression but measures observed counts instead,

$$\text{G-test}(i,j)=2\sum_{a,b}{\text{Obs}}_{ij}^{ab}\text{log}\frac{{\text{Obs}}_{ij}^{ab}}{{\text{Obs}}_{i}^{a}{\text{Obs}}_{j}^{b}},$$

thus introducing an additional dependence on how many symbols are observed in the columns, which is a function of how gappy they are. That is, while two sets of pairs with the same residue probabilities have the same MI regardless of the number of gaps, their G-test will be higher for the pair with fewer gaps.

Thus when the process of generating null alignments does not respect gap structure, and the input alignment has a non-uniform gap structure (as in Figure 3b top) a covariation measure such as G-test can wrongly assign higher scores to pairs because of their higher occupancy, relative to randomized null alignments (Figure 3b bottom left). Null alignments that respect the input alignment gap structure (Figure 3b bottom right) will not fool covariation signals like G-test with extraneous signals from inhomogeneous gap distributions. On the other hand, MI which is agnostic to occupancy, performs similarly in both types of null alignments, and finds no significant covariation in either one of them, but MI is noisier in low occupancy columns, which is also undesirable.

R-scape uses G-test as its default covariation measure. Since version v2.0.0.g and later, R-scape creates null alignments that preserve the position of gaps in the input alignment.

### 2.4 | [4] Beware of what the covariation statistic actually measures

A ‘covariation’ statistic that measures more than just covariation may detect significance when there is no covariation.

There are covariation statistics that measure a combination of covariation, conservation and compatibility with an RNA structure, such as the RNAalifold (RAF) measure^69^ or RNAalifold with stacking (RAFS)^66^ . For every pairwise sequence comparison, the RAF measure has a positive score (+2, +1) for a double and a half-compensatory change respectively. In addition, it has a negative term that penalizes (with score −1 per sequence occurrence) base pairs that are non-Watson-Crick and also base pairs that include at least one gap. By penalizing gaps, they effectively favor conserved pairs. By penalizing non-Watson-Crick base pairs, the statistic favors consistency with an RNA structure.

RAF(S) measures have been used successfully for making RNA alignments, as in the method RAF,^15^ where conservation and consistency are both useful information since the method assumes that the sequences to be aligned are both homologs and structural.

However, RAF(S) measures create artifactual “significant covariation” because of how they interact with the method to estimate null alignments to assess a conserved RNA structure. The random-site sampling method used to place the substitutions of a given evolutionary history does not preserve position-specific sequence conservation in the original alignment. In Figure 3a, we can see that while the original alignment has four completely conserved positions, only two of those have survived in the left null alignment, and none in the right null alignment. Thus, because an RAF(S) statistic rewards a pair of highly conserved positions compatible with a base pair (even with no compensatory substitutions), and R-scape’s null alignments do not preserve position-specific conservation patterns, any statistic that mixes covariation with conservation (such as RAFS^66^) can wrongly assign pairs with zero covariation and no compensatory substitutions at all as “significant”.^70^

**FIGURE 4:**
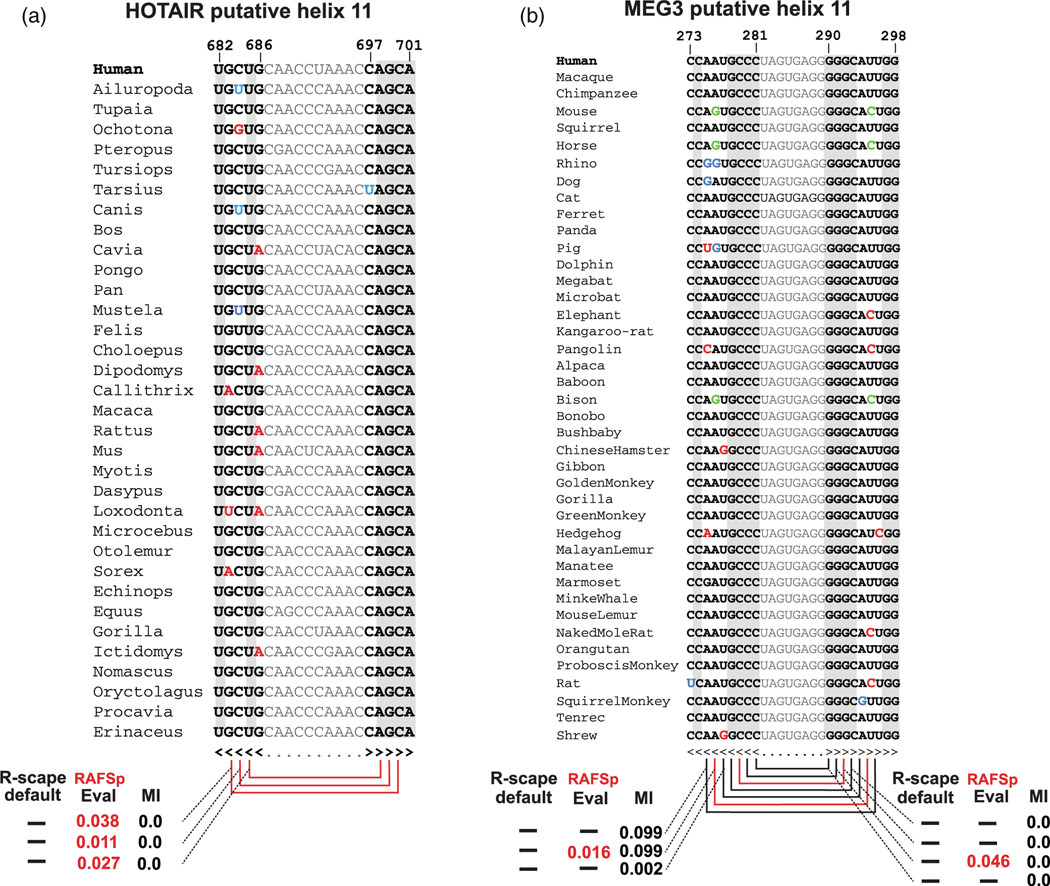
Tavares’ analysis [85] calls pairs of noncovarying conserved primary sequence positions “significantly covarying.” (a) Tavares et al. 37 sequence alignment of HOTAIR Domain 1 putative helix H11 where they show a 5 base pair helix and claim “covariation support” for three base pair (red arcs). The ACG in right-hand side of the pairs is completely invariant, thus by definition, there is no covariation. (Example from,^70^ as observed in Figure 5b of.^71^) (b) Uroda et al. 42 sequence alignment of MEG3 putative helix H11 showing a nine base helix in which they claim covariation support for two base pairs (red arcs) and potential covariation support for five more (black arcs) using the same Tavares’ analysis. Four of the seven base pairs (GCCC-GGGC) show no covariation and no compensatory base pair substitutions, including one pair (C-G) claimed significantly according to RAFS. The other pair claimed “significant” includes three double substitutions, but also four species where the base pair is not preserved, and also minimal Shannon mutual information (MI). Results obtained by running command line R-scape -s -RAFSp (from R-scape v0.8.2) on the provided alignments. Residues in red disrupt the proposed Watson-Crick pair, residues in blue show a half-compensatory substitution, and residues in green show a double-compensatory substitution. Shaded columns are 100% sequence conserved. Coordinates are relative to the given alignments (also provided with this Supplemental Materials).

Figure 4 shows two published examples of this artifact.^71,72^ For the lncRNA HOTAIR domain 1,^71^ proposes a structure with several base pairs deemed to covary according to R-scape using the RAFSp measure (a variant of RAFS that includes an average product correction^65^). A close analysis of a five-base pair helix where three of those pairs are deemed significant shows that in all three cases, the right-hand side of the base pair consists of a completely conserved column, thus lacking any covariation (Figure 4a). A standard analysis with R-scape default covariation measure results in no significant covariation for any pair in the whole HOTAIR D1 alignment. On the other hand, the alignment has sufficient power to expect 21 covarying base pairs, overall suggesting evidence against the presence of a conserved RNA structure.

The same RAFS analysis^71^ has also been used on a proposed structure for the lncRNA MEG3.^72^ Figure 4b shows MEG3 putative helix H11 for which 7 potentially covarying pairs are presented, two of them claimed significantly. A closer analysis of the alignment shows that 4 of the 7 pairs are completely conserved (including one of the two significantly covarying according to RAFS), and the other 3 have minimal MI. A standard analysis with R-scape also results in no significant covariation, but the alignment has sufficient power to expect 35 covarying base pairs.

Since version 1.4.0, R-scape disallows the use of any RAF-related covariation measure to assess statistical significance. We strongly discourage using earlier versions of R-scape in order to apply an RAF(S) measure to the statistical test.

### 2.5 | [5] Beware of machine-learned covariation measures

Models that infer pairwise coupling terms describing the alignment do not automatically provide evidence for a conserved RNA structure.

Models from machine learning and statistical physics with pairwise coupling parameters have been applied to RNA alignments. Amongst those, direct coupling analyses (DCA) based on statistical Potts models^73^ have attracted a lot of attention in recent years, both for the prediction of protein 3D structures,^74–78^ as well as for RNA structure.^79–81^ The advantage of these methods for prediction accuracy of RNA base pairs seems to be limited to non-Watson-Crick pairs.^82^ Importantly, DCA does not separate phylogenetic covariation, and thus does not reliably detect only RNA structural covariation. The R-scape statistical significance test could be applied to DCA covariation scores but the time needed to estimate the parameters makes the approach impractical.

Recent developments in 3D structure prediction using deep learning methods show that adding evolutionary information in the form of an alignment and covariation scores and incorporating both jointly into attention methods^83^ is very good for improving 3D structure determination both for proteins^84^ as well as for RNA.^85^ However, the question of whether there is or is not a conserved RNA structure at all cannot be addressed directly by those methods. Methods for 3D RNA structure prediction are tested on RNA-Puzzles competitions,^86,87^ where it is assumed to begin with that the mystery RNA is structural and conserved, and the emphasis is on the problem of getting the molecule’s spatial coordinates correctly determined.

### 2.6 | [6] Beware of what covariance models measure

Significance with respect to an RNA covariance model (CM) does not guarantee covariation.

An RNA CM is a probabilistic model (a stochastic context-free grammar) that describes a particular conserved RNA structure. The method Infernal,^14^ given a structural alignment, builds a CM model that represents the structure

$${\text{P}}_{\text{first bp (G:C)}}^{\text{RF}0031}=\begin{array}{c} A\\ C\\ G\\ U \end{array}\left(\begin{array}{cccc} A & C & G & U\\ 0.000 & 0.000 & 0.000 & 0.047\\ 0.000 & 0.035 & 0.201 & 0.000\\ 0.000 & 0.348 & 0.000 & 0.123\\ 0.231 & 0.015 & 0.000 & 0.000 \end{array}\right),\;\text{such that}\sum_{a,b=1}^{4}{\text{P}}_{\text{first bp (G:C)}}^{\text{RF}00031}(a,b)=1.$$

and all its expected sequence and structural variability. Infernal CM models can be used to search sequence databases for structural homologs and to align them. CMs measure a mix of covariation, conservation and compatibility with an RNA structure, which are all informative properties for the purpose of homology search and alignment. However, much as the issue with the RAF(S) statistics, a CM score can create artifactual “significant covariation.”

Several publications have used high CM scores as evidence that a conserved RNA structure exists.^44,88,89^ As an example, we concentrate on a filtered subset of 40,078 RNA selected from a screen that predicted over 516,000 conserved vertebrate RNAs structures predicted using CMfinder^44^ believed to be a trusted subset of de novo conserved RNA structures.^89^

We have investigated the subset of 40,078 CMfinder-derived candidates for the presence of a conserved RNA structure. First, because the candidates were selected based on the human-centered 100-way vertebrate UCSC Genome Browser alignment^44^ which is not aware of any structural constraints, we tried to enhance the possible structural signal by making realignments producing Infernal models and creating two additional alignments per candidate, one using the Infernal tool cmalign which simply realigns all the sequences according to the CM, and another one using the Infernal tool cmsearch which only aligns sequences below an E-value threshold to assess homology. Our investigations are based on the combined information provided by the three types of alignments.

A reanalysis using R-scape of the subset of 40,078 CMfinder-derived candidates indicates that only a small fraction of the candidates (2.5%, 1,021/40,078) have evidence of a conserved RNA structure (Table 1). Why do many candidates have good scores, but so few of them have evidence of a conserved RNA structure according to the R-scape approach?

A critical component in a CM is the base pair emission probabilities that assign a probability to the 16 possible pairwise nucleotides.^12^ Each base pair in a given CM gets assigned a different pair emission probability distribution. For instance, the first base pair of the Rfam CM model for the SECIS_1 element (RF00031) is a G:C consensus pair with the following probability distribution (calculated before adding pseudocounts, which are used to avoid the case of zero probabilities),

For this consensus G:C pair, while compensatory double-substitutions such as C:G and A:U (in green) and half-compensatory G:U (in blue) have high probability, still the highest probability corresponds to the actual consensus G:C pair (bold). Thus, the CM is modeling both base pair conservation and well as covariation associated to RNA secondary structure constraints.

Thus, a CM pair emission probability distribution reflects *both the conservation and the covariation* observed for that base pair, which is exactly what you want for the purpose of homology detection, but a confounded measure for detecting structural covariation. A CM will assign high scores simply due to sequence conservation and not to structure conservation.

Indeed, going back to our example, we observe that for more than half of the candidates (53%, 21,204/40,078) the sequences are too conserved to be able to assess whether there is an RNA conserved structure or not (no covariation and no power).

Power is defined as the expected covariation that we should observe if there were a conserved structure, given the amount of variability we observe in the columns. We calculate power by estimating for a collection of structural RNAs the observed covariation as a function of the number of substitutions in the base pairs. No covariation in the absence of power indicates that the alignment has no information to decide on whether a structure is conserved or not. No covariation in the presence of power shows evidence against a conserved RNA structure.

For the collection of 40,078 candidates, almost a third of the candidates (29%, 11,534/40,078) do not have covariation but they have sufficient power (Table 1). We leave a follow-up discussion of the 1,021 candidates with covariation support for Issue 8.

### 2.7 | [7] Beware of covariation due to sources other than RNA structure or phylogeny

Rejecting a null hypothesis (phylogenetic covariation) does not mean that your favorite hypothesis (RNA structural covariation) is true.

We have encountered an unexpected source of pairwise covariation that arises neither from phylogeny nor from RNA structure. In alignments of protein-coding exons, we detect significant covariation between the positions in a given codon (within-codon covariation). Covariation between adjacent positions cannot be due to Watson-Crick RNA base pairs as those are required to be separated by at least three unpaired nucleotides. (Incidentally, there are non-Watson-Crick adjacent pairs, but those are few and do not show much covariation.)

Using alignments for all intronless protein-coding genes in *S. cerevisiae*, we observe that overall 38.5% of all the significant covariations are within-codon covariation. That is in contrast with RNA structural covariations which are predominantly distant (95% of covariation in *S. cerevisiae* structural ncRNAs are more than three nucleotides apart).^91^ Other studies have also reported coding covariations.^92^ Significant covariation between adjacent or next to adjacent positions is a signal indicative of protein-coding exons, not yet exploited, that could be incorporated into methods to detect protein-coding exons from alignments.

Within-codon covariation is the result of both synonymous and non-synonymous codon substitutions.

Here we show that the within-codon covariation signal in protein-coding exons arises from constraints of the genetic code resulting from a combination of codon preferences and amino acid evolution.

**FIGURE 5:**
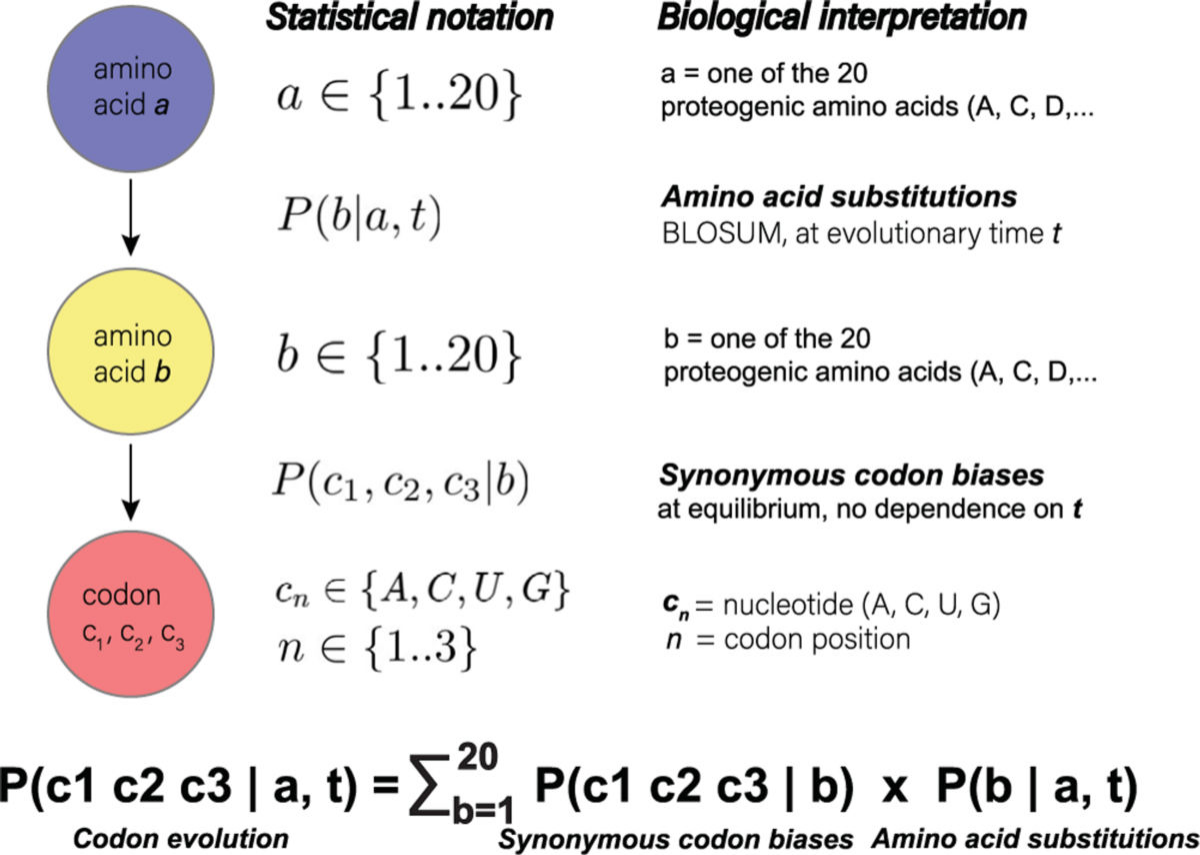
Model of protein-codon evolution. Our model of protein evolution starts with an ancestral amino acid. The probability of amino acid a being substituted by amino acid b is calculated using a BLOSUM matrix at a specified value of evolutionary time t. In the case of nonsynonymous substitutions, a≠b. In the case of synonymous substitutions a=b. Finally, the codon is determined based on amino acid b’s codon biases. It is reasonable to assume that synonymous substitutions (represented by codon biases per amino acid) reach equilibrium quickly, while nonsynonymous substitutions keep evolving for much longer times. Thus, the model makes the approximation of using stationary (no time dependence) codon biases probability distributions

Figure 5 describes a simple model of protein-codon evolution in which the probability of observing a codon c1c2c3 at time t resulting from an ancestral amino acid a can be computed by combining a standard amino acid substitution matrix with standard codon biases as,

$$P(c1c2c3|a,t)=\sum_{b=1}^{20}P(c1c2c3|b)P(b|a,t).$$

In this evolutionary model, the first term describing codon biases accounts for one source of variation. Biologically, we use them to represent synonymous substitutions, which are substitutions that do not alter the amino acid. Most synonymous substitutions only involve variation at the third codon position (referred to as wobble) as in the case of cysteine, which can be encoded with either U or C at the third position so long as the first and second positions are fixed as U and G. However, the three 6-box amino acids (leucine, arginine, and serine) can undergo synonymous mutations that also alter the first and/or second codon positions.

The second term describes amino acid substitutions, the second mechanism of protein evolution in our model. In contrast to synonymous substitutions, nonsynonymous substitutions can greatly change the codon’s nucleotide composition as it is no longer restrained to encode the same amino acid. This allows it to theoretically become any of the other 63 codons. Biologically, however, it has been observed that some amino acid substitutions are much more common than others. This is because changing the amino acid may result in deleterious changes to protein structure and function. Thus, it has been observed that amino acid substitutions tend to favor changes that maintain similar chemical properties such as polarity, charge, and shape. To describe the likelihood of an amino acid either staying the same (synonymous substitution) or mutating into another amino acid (nonsynonymous substitution), matrices have been computed using real protein alignments. These matrices can be adjusted with respect to a parameter t that represents a unit of evolutionary time.

While our model involves both synonymous and nonsynonymous substitutions, this simplified model ignores insertions and deletions. Nor does it consider nonsense mutations, which would change a sense codon to a stop codon probably worth including, but the amino acid substitution models like BLOSUM do not consider sense-to-stop substitutions (or vice versa), which is why our model does not either. It also assumes that all sequences in the alignment are independently derived from the ancestral sequence (a star topology).

**FIGURE 6:**
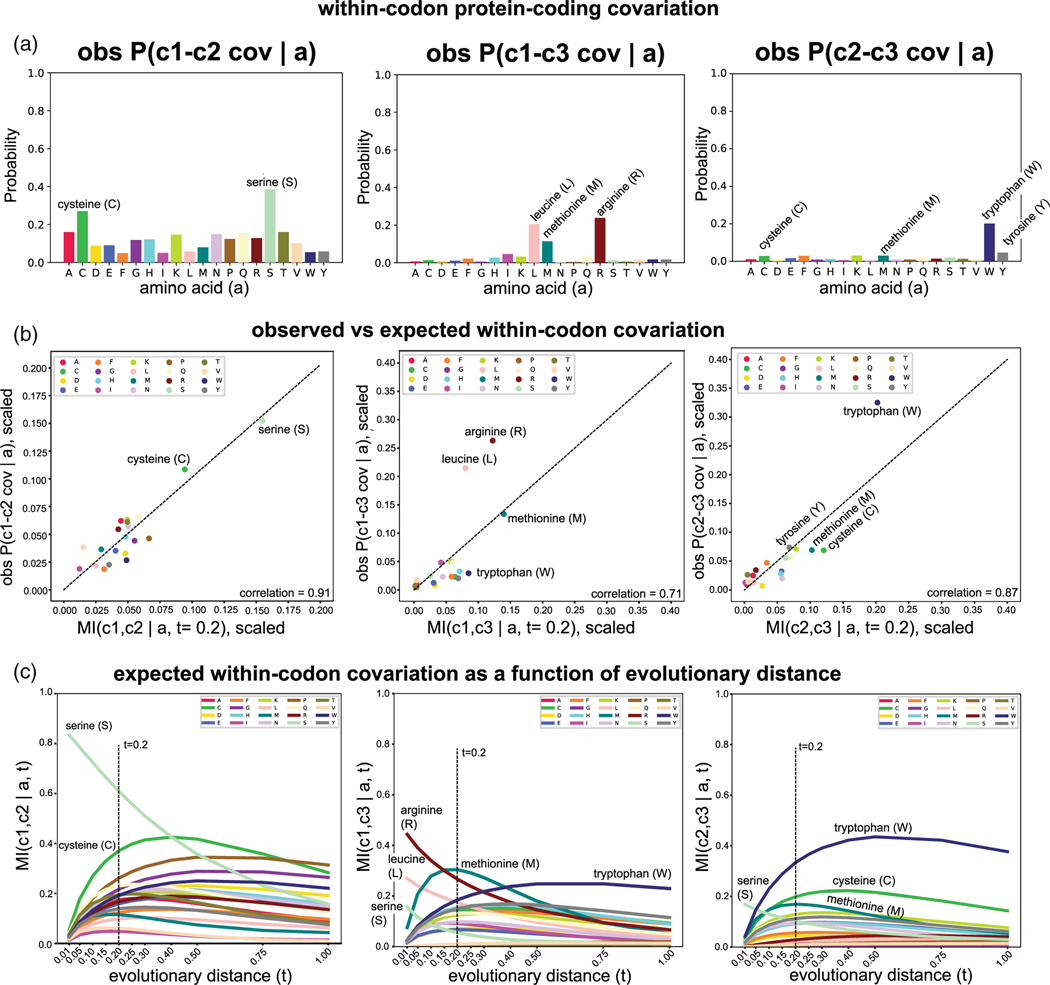
Within-codon protein-coding exon covariation. For the 4,826 annotated intronless protein-coding genes in *S. cerevisiae*, we generated alignments using nhmmer^93^ (E-value <1.0 × 10^10^) searches of a database of 1,371 genomes of the *Ascomycota* fungal phylum.^47^ (a) The observed fraction of codons for which the c1-c2 (or c1-c3 or c2-c3) codon positions significantly covary (R-scape E-value <0.05) conditioned on the corresponding amino acid (as represented by the *S. cerevisiae* aligned codon). (b) Comparison of the observed within-codon covariation per amino acid to the expected covariation (measured by MI) given the model of evolution. For amino acid substitutions, we use a rate matrix derived from BLOSUM62 normalized to one substitution per site. We use the evolutionary divergence of t=0.2 which produces on average alignments with average percentage identity of 58% similar to that observed on average for the intronless protein-coding alignments. Both the observed frequency of within-codon covariation and the expected mutual information are scaled to a total sum of one, just for better visualization. (c) Description of the expected (unscaled) MI for within-codon covariation as a function of divergence time. Trajectories that monotonically decrease with time correspond to the three amino acids (serine, arginine and leucine) decoded by 6 different codons. Arginine (CGN, AGR) and leucine (UUR, GUN) allow synonymous changes between the c1 and c3 codon positions, and the c2 position is fixed (middle panel), and serine (UCN, AGY) allows all three types (c1-c2, c1-c3 and c2-c3) of synonymous changes

Figure 6a describes the amount of within-codon covariation (c1-c2, c1-c3 or c2-c3, where c1, c2, c3 are the codon positions) observed for the different encoded amino acids. We observe that a small set of amino acids drives each of the three different cases.

Using the evolutionary model in Figure 5, we can calculate the expected within-codon covariation as the MI of the corresponding marginal probabilities, and we can compare those to the within-codon covariation observed in the yeast exon alignments. We present those correlations in Figure 6b where the expected covariation has been calculated at t=0.2 which corresponds to the average pairwise identity (58%) found in the yeast intronless alignments. We observe that there is a good correlation between observed and expected covariation given the evolutionary model of Figure 5.

Figure 6c describes more generally the predicted evolution of within-codon covariation as a function of evolutionary divergence given the model. We observe that for short evolutionary divergence, within-codon covariation is dominated by the three amino acids coded by 6 different codons. As those are the only amino acids that support synonymous changes in all codon positions, thus they are the only cases that in the absence of non-synonymous substitutions can result in within-codon covariation. As divergence increases, non-synonymous substitutions become more prevalent and within-codon covariations are spread more regularly amongst the different amino acids. In particular, amino acids that are restricted in genetic code usage (methionine, tryptophan) or have strong evolutionary constraints per the BLOSUM matrix (cysteine, tryptophan) have the highest within-codon covariation at moderate evolutionary divergences.

mRNA-induced covariation can coexist with RNA structural covariation.

That is the case of the transfer-messenger RNA (tmRNA), a bacterial structural RNA that also includes a protein-coding sequence. When an mRNA lacking a stop codon gets stalled at the ribosome during translation, tmRNAs provide an mRNA template ending with a stop codon that frees the ribosome from the defective mRNA. The mRNA template sequence is also part of the structure making half of an RNA base-paired helix. In alignments of tmRNAs from different bacteria, R-scape can find non-phylogenetic covariation that can be easily associated either to the hairpin elements or to codon structure.^34^

### 2.8 | [8] Beware of structural fragments

Sometimes a structural RNA may not be functional.

One such case is the nuclear mitochondrial DNA sequences (NUMTs) which are inserted in nuclear genomes in multiple copies including mitochondrial rRNA and tRNA fragments and show evolutionary conservation in the nuclear genome.

Within the 40,078 proposed conserved human RNA structures of Ref. 89, we identify a subset of 1,021 with covariation support (at least 3 covarying base pairs in all three alignments). Further analysis indicates that only 71 of them do not show any similarity to an already known Rfam family. Those similarities are mostly fragments of rRNA (137 LSU, 117 SSU), tRNA (371), snoRNAs (65) and miRNAs (82) (Table 1).

The remaining 71 candidates would then appear to have high potential of being novel conserved structural RNAs. But further analysis indicates that many of them are homologous to fragments of mitochondrial tRNAs and rRNAs. These inserts named NUMTs do not seem to have any specific function. NUMTs are well-known and present in all vertebrate species.

As an aside, we finally identify five of the 40,078 proposed structures with enough potential to be structural RNAs (those are: cmf.M0662940, cmf.M0914129, cmf. M0950988, cmf.M2247904, cmf.M2299369).

### 2.9 | [9] Beware of pseudogenes

Pseudogenes increase apparent power but dilute covariation signal.

There is an astounding proliferation of pseudogenes derived from ncRNAs.^94^ Those are sequences related to a given ncRNA that show evidence of not performing the ncRNA function, either because of their fragmented or repetitive nature. Examples of human ncRNA pseudogenes of both kinds are the hundreds of 5 S rRNA-related sequences^95^ or the SRP-derived Alu interspersed repeated elements, respectively.

Pseudogenes derived from ncRNAs retain both sequence and structural similarity, and they are often difficult to distinguish from the functional ncRNA. Indeed many pseudogenes show up in homology searches both structural or just sequence-based. Because substitution in pseudogenes is not structurally constrained, they can appear at every position, and they will slowly degrade any evolutionary constraint of the functional ncRNA. Thus, alignments of structural ncRNAs that include pseudogenes will tend to show an overall increased power of covariation together with a decrease of covariation at base-paired positions. We propose that in genomic searches for novel structural RNAs that result in many hits per genome, only should only retain the top-scoring sequence per genome because that sequence is more likely to be the true ortholog rather than a confounding pseudogene.

**FIGURE 7:**
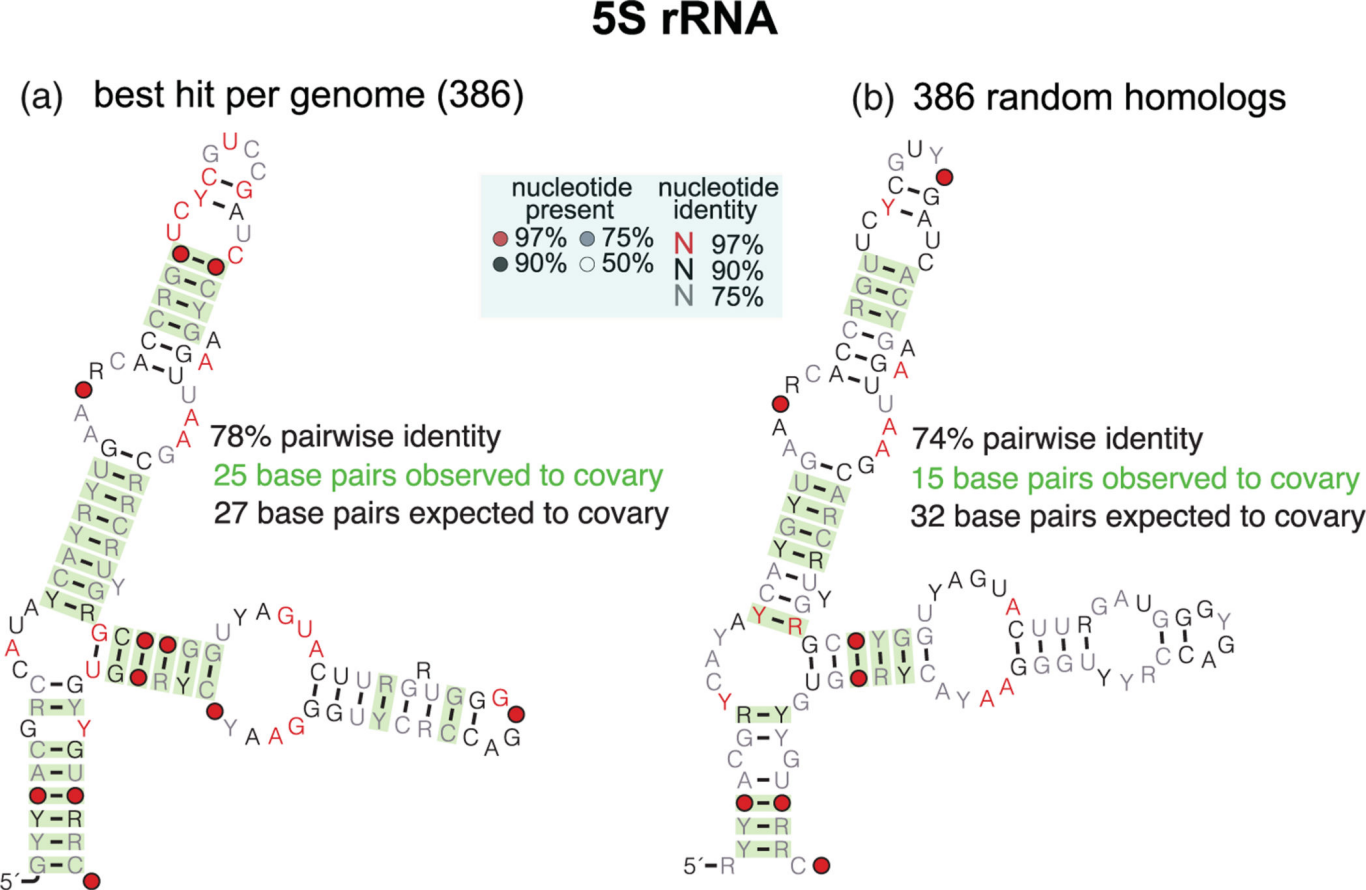
Pseudogenes increase power but reduce covariation. Invertebrate 5 S rRNA sequences were selected and aligned using Infernal (cmsearch) and the 5 S rRNA Rfam model (RF00001) against the NCBI database of invertebrate genomes. We identified 97,352 5 S rRNA homologs with E-value <1 × 10^10^ from 386 different invertebrate genomes. **(a)** Sub-alignment of 386 sequences using only the best hit per genome. **(b)** Sub-alignment of 386 5 S rRNA-related sequences selected at random amongst all significant hits. Evolutionary analysis of observed and expected covarying pairs performed using R-scape with option -s that evaluates how well a proposed consensus structure is supported by covariation

As an example, we used the Rfam model for 5 S rRNA (family RF00001) to search a database of invertebrate whole genomes from NCBI. We identified 97,352 homolog sequences (with E-value <1 × 10^10^) in 386 genomes. *D. melanogaster* has 96 5 S rRNA-related sequences, and *Andricus curvator* (a gall wasp) the species with the most homologs includes over 1,200 homologs. A comparison of the evolutionary support for the 5 S rRNA structure alignments with and without pseudogenes is given in Figure 7. An alignment including only the best scoring sequence for each genome, likely to include true orthologs, results in 25 covarying base pairs (for 27 pairs expected to covary), out of the 38 base pairs in the 5 S rRNA structure. However, an alignment with the same number of sequences but sampled at random from the whole alignment, likely including pseudogenes, reduces the number of covarying pairs to 15, while the expected number of covarying pairs rises to 32.

### 2.10 | [10] Beware of spurious covariation induced by misalignments

Misalignments of RNA helices with variable number of base pairs can induce spurious covariation when both base-paired residues for one sequence are shifted simultaneously to wrong base-paired positions within the helix.

**FIGURE 8:**
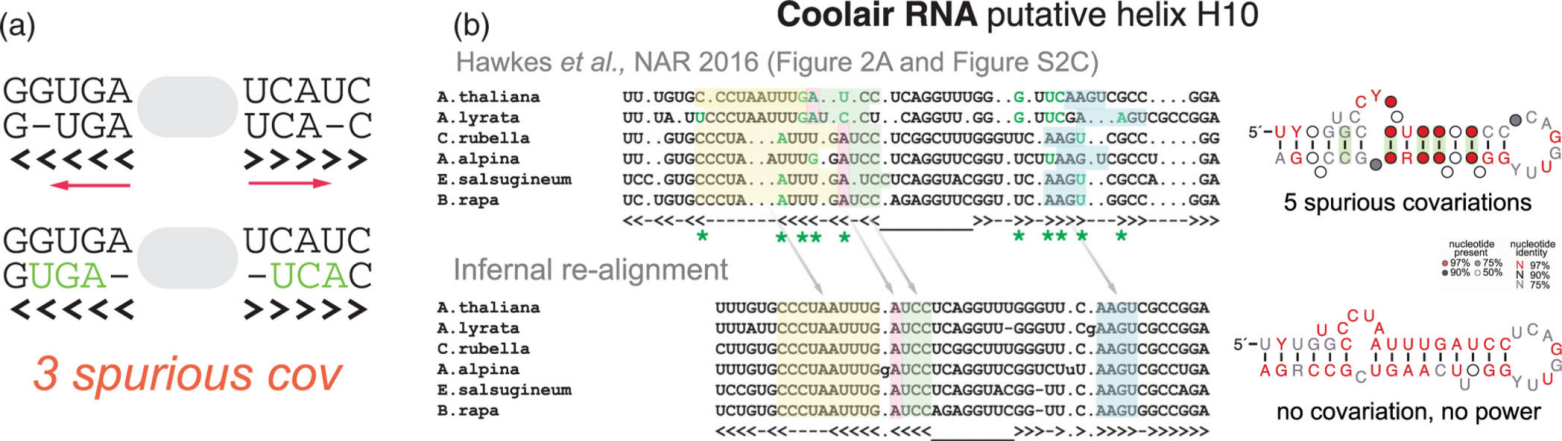
Base pair misalignments induce spurious covariation. (a) Misalignment mechanism that results in spurious covariation at the expense of reducing sequence conservation while preserving the same structure. (b) Example of the artifact from a proposed structure for the lncRNA COOLAIR.^96^ Sequences structurally realigned using Infernal show a completely conserved putative helix with no covariation support. Realigned groups of residues are equally colored in both alignments.

We have reported elsewhere^82^ how misalignments can result in spurious covariation. Those results are because base pair stems can have different numbers of base pairs in different species (Figure 8a).

An example is shown in Figure 8b. An alignment of the lncRNA COOLAIR appears to have covariation,^96^ but an automatic realignment shows a more conserved arrangement that increases sequence conservation while preserving all the proposed Watson-Crick base pairs, thus providing no evolutionary information as to whether there is an evolutionarily conserved structure for COOLAIR or not.

### 2.11 | [11] Beware of spurious covariation induced by non-homologous sequences

Paraphrasing Karlin & Altschul,^97^ high-scoring structural alignments of non-homologous sequences will be pressured to resemble the proposed structure which can force spurious covariation.

The detection of homologs either by sequence alone (BLAST, nhmmer) or sequence and structure (Infernal) relies on the characterization of the distribution of scores of non-homologous sequences. High-scoring non-homologous sequences will necessarily resemble the motif under consideration. In particular, for structural homology the inclusion of non-homologs will tend to force the alignment to introduce substitutions to fit the consensus structure and these will result in apparent covariation. This effect will be exacerbated when the sequence of interest has low complexity (such as including large runs of a repeated nucleotide, as a homopolymer of A nucleotides will appear to base pair with a homopolymer of Us).

Thus, when analyzing the evolutionary signal in structural alignments, it is important to avoid the inclusion of non-homologous sequences. And it is safer to use sequence alignment alone, as opposed to risking the circularity inherent in performing an alignment that maximizes structural similarity, and then evaluating the support for that structure.

**FIGURE 9:**
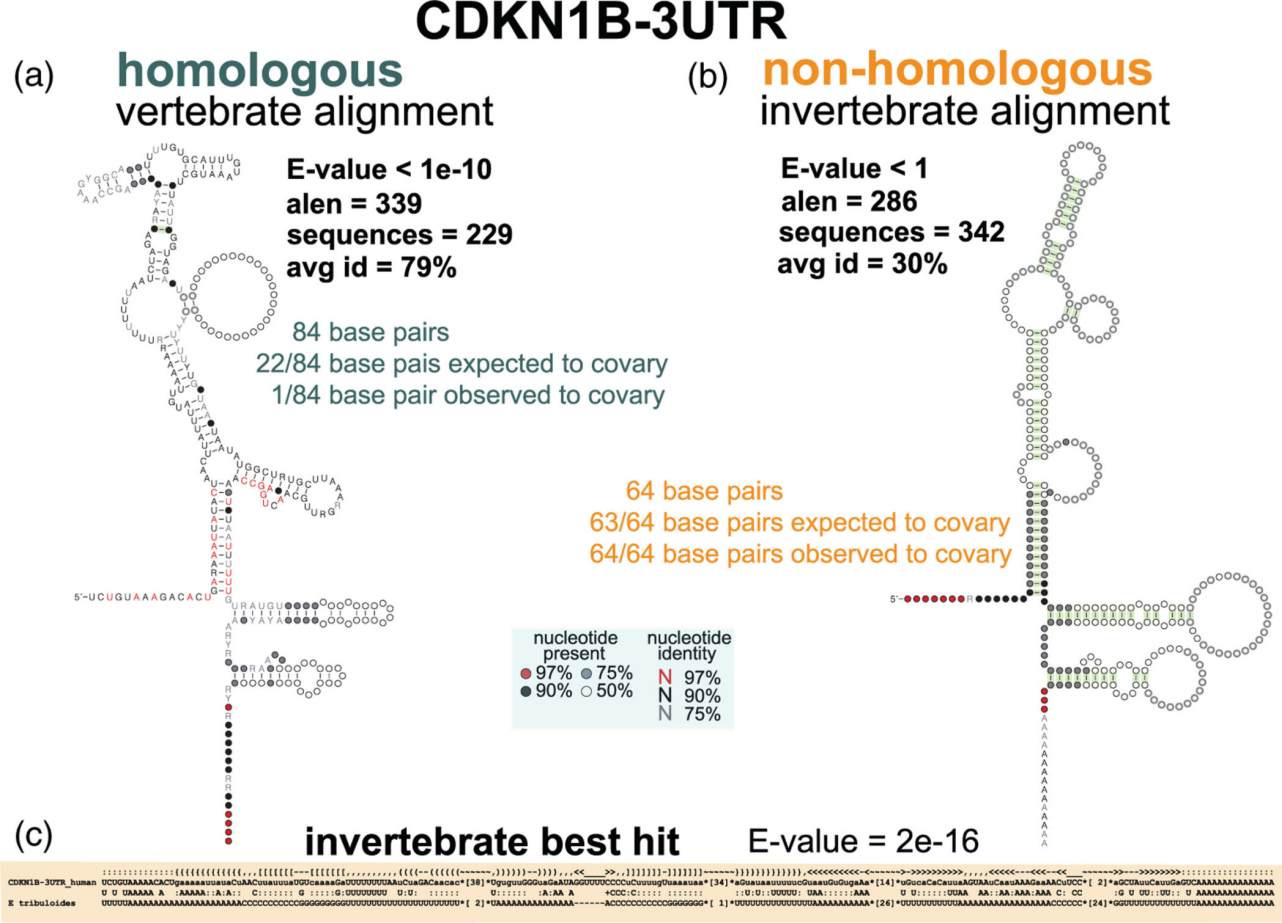
Structural alignments of non-homologous sequences can introduce spurious covariation. An Infernal model of the human CDKN1B-3′-UTR is used to search a database of (a) vertebrates and (b) invertebrate genomes. We show the evolutionary analysis of the two alignments. (c) Alignment to the Infernal model of the best scoring invertebrate hit. The proposed structure is given in WUSS format (see Infernal documentation for a description of the WUSS structural notation)

As an example, Figure 9 shows the 3′-UTR of the human CDKN1B gene for the p27 cyclin-dependent kinase inhibitor. The CDKN1B-3′-UTR is conserved in vertebrates. A structural alignment constructed using Infernal and using a proposed structure for the motif is presented in Figure 9a. This vertebrate alignment is likely to include real homologous sequences, but it does not provide much convincing evidence for the proposed structure to be conserved. Figure 9b, which uses a larger E-value cutoff (E < 1 vs. E < 1 × 10^10^), shows an invertebrate structural alignment with similar number of sequences, all non-homologous sequences. This invertebrate alignment has very low % average pairwise sequence identity, and all proposed base pairs appear to covary! Figure 9c shows the highest scoring invertebrate sequence in this alignment (E-value 2 × 10^16^), and how by visual inspection it is obviously a non-homolog because it is a repetitive sequence that even looks like a sequencing artifact (ordered runs of A, C, G, and Us). The alignment, by being forced to conform to the proposed structure, introduces a striking number of spurious covariations.

### 2.12 | [12] Beware of diverse evolutionary rates in structural RNAs

Structural RNAs have a wide range of different evolutionary rates. The covariation evidence for evolutionary conservation of their consensus structure requires us to use comparative data at the optimal evolutionary distance.

Different known structural RNAs have many different evolutionary rates, and in order to identify them, we need to find alignments with enough sequence divergence to capture the covariation signal. This effect is exacerbated when searching for structural RNAs in vertebrates due to the much shorter evolutionary distances between the existing sequenced vertebrate genomes in comparison with those existing between bacterial or archaea genomes. For slowly evolving structural RNAs such as tRNAs or rRNAs, alignments restricted to vertebrates usually have too little variability to detect any signal of evolutionary conservation. On the other hand, structures such as the iron response elements (IRES)^98^ or the selenocysteine insertion sequences (SECIS)^99^ both found in vertebrate UTRs are short (less than 70 nts) and can go undetected if the homology search is not well tuned to their evolutionary divergence.

**FIGURE 10:**
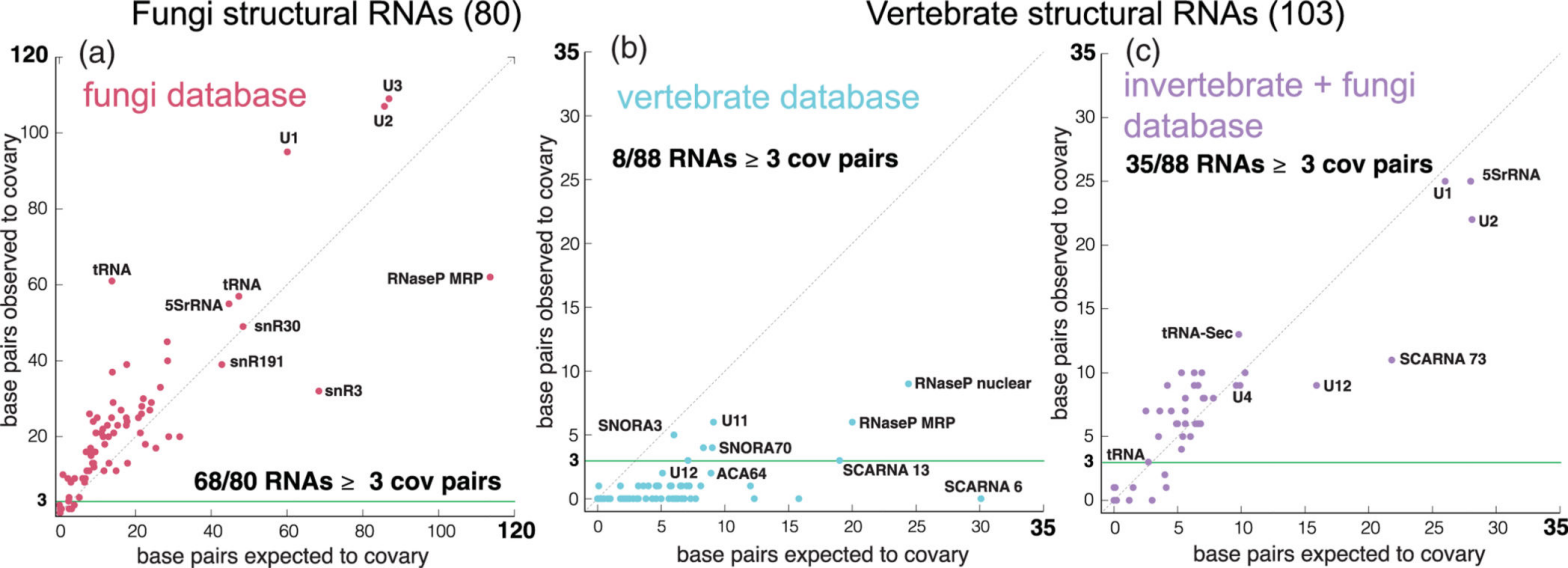
Evolutionary signal for known conserved structural RNAs in eukaryotes; comparison between fungi and vertebrates. We show control screens in which a set of known structural RNAs in some genome (*S. cerevisiae* and *T. rubripes*) are used to search a genomic database with a homology method (nhmmer) to find homologs, build an alignment and evaluate the potential for a conserved RNA structure with R-scape. (a) Control screen of 80 known structural ncRNAs in the budding yeast *S. cerevisiae* using a database of 1,371 complete fungal genomes.^47^ Each RNA is represented by the observed number of significantly covarying base pairs versus the expected number of covarying pairs. We find that 69/80 RNAs have at least 3 covarying base pairs. (b) Control screen of 103 known structural ncRNAs in pufferfish (*T. rubripes*) compared to a database of 183 vertebrate genomes which yielded alignments for 88/103 RNAs. Only eight of the 88 RNAs have at least three covarying pairs. (c) Same 103 RNAs compared to a combined database of 969 invertebrate and 1,371 fungal genomes which also yielded 88 alignments. 35/88 RNAs have at least 3 covarying base pairs. The vertebrate genomes were selected by clustering the collection of NCBI vertebrate genomes by the similarity of their RNaseP RNA, and taking a representative from each cluster (genomes in a cluster have at least 85% similarity to each other’s RNaseP RNAs).^100^ In all cases, we constructed multiple sequence alignments through three rounds of homology search (using nhmmer) at E-value cutoffs of 1 × 10^−10^, 1 × 10^−10^, 1 × 10^−5^, respectively

We have performed screens in the yeast *S. cerevisiae* and the pufferfish *T. rubripes*, in which regions in the genome of interest are compared using nhmmer to a database of whole genome sequences, and the resulting alignment of homologs is evaluated for structural covariation using R-scape. Figure 10 shows controls for such screens using known structural RNAs either in yeast or pufferfish, using the same screen details,^47^ and different comparative data. We observe that while a large fraction (85%) of the fungal structural RNAs, when compared to a database of fungi genomes, would be detected (as having at least 3 significantly covarying base pairs), only a small fraction (8%) of the pufferfish structural RNAs would be detected searching in a database of vertebrate genomes.

We also observe how a comparison to a database of invertebrate and fungi genomes increases performance in identifying known structural RNAs (24%). The reason for this is that most of the tested structural RNAs are slowly evolving and they have very high sequence similarity when restricted to vertebrate genomes alone.

As a fungi example, the yeast RPS2 5′-UTR structure that we re-identified in the fungi screen^47,101^ with only 40–50 nts, barely produced alignments in the first and second iteration (E < 1 × 10^10^), and only in the third iteration ((E < 1 × 10^5^) there was enough variation to detect covariation. And additional screens starting from four different fungi species (*C. albicans, N. crassa, A. fumigatus*, and *S. pombe*) expanding three different *Ascomycota* subphyla resulted in the discovery of different structural RNAs which were not found in the other subphyla.^47^

As vertebrate examples, the IREs and SECIS structural RNAs both present in the pufferfish positive control did not report any homologs in an nhmmer search performed with E-values (<1 × 10^10^. In addition, using the UTR of the human GPX1 gene that includes a SECIS structure, an nhmmer search against a vertebrate database tuned to the evolutionary divergence of the structural RNaseP RNA, and using larger E-values <1 results in an alignment of 156 vertebrate sequences with 61% identity, but it does not report any covarying base pairs (alignment is provided in the supplemental material).

Different sets of comparative genomes need to be used to optimally detect structural RNAs at a varied range of evolutionary rates.

### 2.13 | [13] Beware of when the joint power of a base pair is not consistent with the sum of each position’s power

Inconsistent single versus double substitution power reflects lack of base pairing evidence.

**FIGURE 11:**
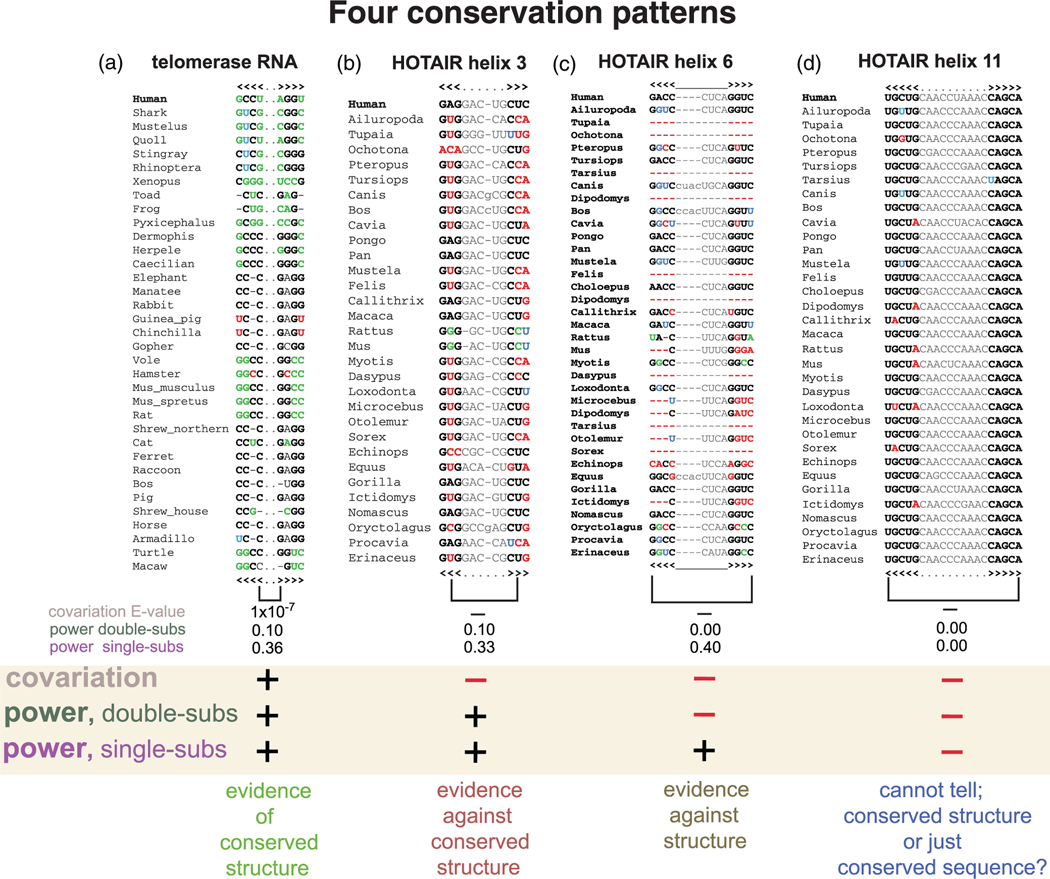
Four different conservation patterns and their implications for inferring conserved RNA structure. (a) Detail of the vertebrate telomerase RNA Rfam seed alignment (RF00024). (b) From HOTAIR domain1, putative helix 3 from the alignment provided by Somarowthu et al.^102^ (c) From the same HOTAIR domain1 alignment, putative helix 6. (d) HOTAIR domain1, putative helix 11. R-scape report power by single substitutions by default. Option –doublesubs report power by double substitutions

In the calculation of statistical power, the default is to count the number of substitutions observed in both individual columns and sum them.^32^ This default calculation assumes that the variation is shared more or less equally between the two columns. Alternatively, we can calculate the number of substitutions that occur in both positions simultaneously. Alignments that do not share the variation between the two columns, or alignments that do not even share the presence of both residues in both positions will lead to disparate results between these two different ways of estimating power. We show an example in Figure 11c.

Four alignment patterns, implications for inferring RNA structure.

We have discussed two computational measures for extracting the evolutionary information contained in an alignment with respect to the question of how to identify conserved RNA structure. One measure is the pairwise co-dependence above what would be expected if there were no conserved structure (**significant covariation**). We say that a base pair significantly covaries if it has an R-scape E-value <0.05, by default. The other measure is how much significant covariation to expect if there were a conserved structure given the sequence variation observed in the alignment (**power**). The power of a base pair is the probability that the base pair is expected to be detected as significantly covarying based only on the variability that is present in the alignment.

Figure 11 describes four possible scenarios based on significant covariation and power.

Significant covariation (covariation for short) necessarily should grow linearly with power (almost in a 1:1 ratio), but power can exist in the absence of covariation. Covariation indicates the presence of a conserved RNA structure (Figure 11a). But in the absence of covariation, three different scenarios can be expected based on power.

The variation observed for a pair of positions can be measured either by counting substitutions per position (single-subs power) or by measuring double substitutions (double-subs power). Double-subs power implies single-subs power, but the reciprocal is not true. Lack of covariation in the presence of double-subs power indicates evidence against an evolutionarily conserved structure (Figure 11b). Moreover, lack of covariation in the presence of single but not double subs power tends to indicate an alignment that does not support the proposed structure (Figure 11c). Finally, lack of covariation in the absence of any power implies that the alignment cannot provide any information regarding the presence or not of a conserved RNA structure (Figure 11d).

## 3 | FUTURE DIRECTIONS

As in the field of matrix exponentials,† we expect that 25 years from now the field of computational methods for detecting structural RNAs will mature in important directions. Hopefully, much earlier than in 25 years, we will have a complete list of the structurally functional RNAs currently hiding in the extensive vertebrate transcriptional landscape. To achieve this goal, we need to develop more sensitive methods that without sacrificing specificity can identify the signals beyond covariation associated with conserved RNA structure. Importantly, the main source of artifacts is the quality of the alignment. Can we ever be sure that an alignment gives us the correct information as to whether the RNA has a conserved structure? More accurate models to perform structural alignments promise to be an important source of progress in the field.

Often thousands of lncRNAs are assumed to be fundamental regulators based on observations in a few specific ones. It seems prudent not to treat lncRNAs as a homogeneous class, and it seems fruitful to use stringent evidence to discern the wide variety of different things that different lncRNAs may be involved in. Those include cases where the act of transcription (rather than the RNA) seems to be the effector, cases where an RNA may work by binding proteins or other RNAs, cases of undetected protein-coding mRNAs, and cases of accidental and non-functional transcripts, none of which needs to involve conserved RNA structure. In addition, we expect a possibly small subset of lncRNAs to exert specific functions through conserved RNA structure. R-scape is a powerful statistical test to discern structural lncRNAs from other lncRNAs in exploratory comparative data analyses. It is our perspective that identifying vertebrate lncRNAs with genuinely conserved RNA structure would lead to the discovery of novel functions of RNA.

## Supplementary Material

supplementary_material

## Figures and Tables

**TABLE 1:** Evolutionary analysis of the 40,078 conserved RNA structures from Seemann et al., (2022)

		From all three alignments			From structural		
		
Alignment	Zero cov pairs	Cannot tell	Negatives	Structural	No Rfam hit	No NUMTs	Final set
UCSD 100-way	26,111 (65%)	21,204 (53%)	11,534 (29%)	1,021 (2.5%)	71	6	5
Infernal (cmalign)	27,298 (68%)						
Infernal (cmsearch)	31,068 (78%)						

*Note*: This subset was selected by Seemann et al. because their sequence is conserved in at least three species (human, rhesus macaque and mouse) with an average sequence length ≥ 80 nts and the human sequence has ≥100 nts, and because their 100-way vertebrate UCSC Genome Browser alignment^90^ has a good CMfinder score.^20^ We provided results for three different types of alignments: the original alignments, and two realignments of the sequences to an Infernal model created for the human sequence using either the program cmalign or cmsearch with E-value 1 × 10^−3^ which sometimes does not include some of the sequences in the alignment.^14^ The no Rfam hit candidates are obtained after removing all hits to Rfam using the Infernal program cmscan. The NUMTs candidates are identified as having homology to the human mitochondrial genome using nhmmer. The final set of five (cmf.M0662940, cmf.M0914129, cmf. M0950988, cmf.M2247904, cmf.M2299369) results after removing one candidate (cmf.M2304242) that appears to be a protein-coding exon. *Definitions*: CANNOT TELL = at least one of three alignments: (expected cov ≤5) / no cov. NEGATIVES = at least one of three alignments: (expected cov ≥8)/no cov. STRUCTURAL = three alignments have at least three covariations. Note that the sum of the three categories CANNOT TELL, NEGATIVES and STRUCTURAL does not need to add up to the total.

## Data Availability

All alignments and data created for this article are provided as part of the supplemental materials, except for the 4,826 intronless gene alignments produced for Figure 6a. Instead, we provide a one-gene example with a tutorial on how to run the scripts. For the 40,078 Seemann et al. 2022 proposed structures, we only provide the data for the subset of 1,021 proposed candidates with covariation support.

## Box

Thirteen Issues to beware of [1] Beware of single-sequence analyses Single-sequence methods (experimental and computational combined) are modestly informative of possible structures, but cannot determine if the structures are conserved. [2] Beware of phylogenetic covariation Phylogenetic covariation is pervasive in all alignments and confounds structural covariation. [3] Beware of what the null hypothesis destroys Any property of the input alignment that the null alignments break (by some shuffling procedure, for example) can lead to spurious significance if the covariation measure depends on it. [4] Beware of what the covariation statistic actually measures A statistic (such as RAF or RAFS) that combines covariation with other signals cannot be used to assess the significance of the structure; the statistic can be driven by the other signals (such as sequence conservation). [5] Beware of machine-learned covariation measures They do not automatically provide assurance of structural covariation. [6] Beware of what covariance models (CMs) measure Significance with respect to a CM does not guarantee covariation, good alignments and homology E-values can occur just due to sequence conservation and consistency with an RNA fold. [7] Beware of covariation due to sources others than RNA structure or phylogeny Rejecting the null hypothesis of phylogenetic covariation does not automatically validate the structural RNA hypothesis. Alignments of protein-coding exons have covariation within codon positions induced both by synonymous and non-synonymous substitutions. [8] Beware of structural fragments resulting from horizontal transfer A conserved RNA structure may not indicate a functional RNA, for instance, the nuclear mitochondrial RNAs (NUMTs) derived from mitochondrial tRNAs or rRNA. [9] Beware of pseudogenes Pseudogenes can reduce the covariation in an alignment. [10] Beware of spurious covariation induced by misalignments Alignment methods that favor covariation over conservation can create spurious covariation. [11] Beware of spurious covariation induced by non-homologous sequences - Structural alignments that include non-homologous sequences can create spurious covariation. [12] Beware of diverse evolutionary rates in structural RNAs It may be a challenge to identify sequenced genomes at the right evolutionary distance for the detection of some structural RNAs. [13] Beware of structural alignments with inconsistent base pair power - When the power of a base pair is not consistent whether calculated by double substitutions or by the sum of single substitutions, that suggests evidence against a conserved base pair.

## Footnotes

* “Nineteen dubious ways to compute the exponential of a matrix”^48^

† “Nineteen dubious ways to compute the exponential of a matrix, twenty-five years later”^103^

## Abbreviations:

BLAST: basic local alignment search tool
CaCoFold: cascade variation/covariation constrained folding algorithm
CM: covariance model
CMfinder: a covariance model based RNA motif finding algorithm
DAC: direct coupling analysis
E-value: expected value
G-test: goodness-of-fit test
HOTAIR: an antisense intergenic long ncRNA of the HOXC locus
Infernal: inference of RNA alignments
IRES: iron response elements
lncRNAs: long non-coding RNAs
LSU rRNA: large subunit ribosomal RNA
MI: mutual Information
mRNA: messenger RNA
miRNA: micro RNA
ncRNAs: non-coding RNAs
nhmmer: nucleotide biosequence analysis using profile hidden Markov models
NUMTs: nuclear mitochondrial RNAs
R-scpae: RNA structural covariation above phylogenetic expectation; RAF, RNAalifold
RAFS: RNAalifold staking
Rfam: database of RNA families
rRNA: ribosomal RNA
SECIS: selenocysteine insertion sequences
snoRNA: small nucleolar RNA
SSU rRNA: smal subunit ribosomal RNA
tRNA: transfer RNA
tmRNA: transfer-messenger RNA
WUSS format: Washington University Secondary Structure format

## References

[1] Winker S, Overbeek R, Woese CR, Olsen GJ, and Pfluger N. An automated procedure for covariation-based detection of RNA structure. ANL-89/42, 1989.

[2] Holley RW, Apgar J, Everett GA, Structure of a ribonucleic acid. Science. 1965;14:1462–1465. 10.1126/science.147.3664.1462 14263761

[3] Gutell RR, Weiser B, Woese CR, Noller HF. Comparative anatomy of 16 S-like ribosomal RNA. Prog Nucl Acids Res Mol Biol. 1985;32:155–216. 10.1016/s0079-6603(08)60348-7 3911275

[4] Noller HF, Kop JA, Wheaton V, . Secondary structure model for 23 S ribosomal RNA. Nucl Acids Res. 1981;9:6167–6189. 7031608 10.1093/nar/9.22.6167 PMC327592

[5] Cech TR, Damberger SH, Gutell RR. Representation of the secondary and tertiary structure of group I introns. Nat Struct Biol. 1994;1:273–280. 7545072 10.1038/nsb0594-273

[6] Pace NR, Smith DK, Olsen GJ, James BD. Phylogenetic comparative analysis and the secondary structure of ribonuclease P RNA—A review. Gene. 1989;82:65–75. 2479592 10.1016/0378-1119(89)90031-0

[7] Akmaev VR, Kelley ST, Stormo GD. Phylogenetically enhanced statistical tools for RNA structure prediction. Bioinformatics. 2000;16:501–512. 10980147 10.1093/bioinformatics/16.6.501

[8] Gutell RR, Power A, Hertz GZ, Putz EJ, Stormo GD. Identifying constraints on the higher-order structure of RNA: Continued development and application of comparative sequence analysis methods. Nucl Acids Res. 1992;20:5785–5795. 1454539 10.1093/nar/20.21.5785 PMC334417

[9] Knudsen B, Hein J. Pfold: RNA secondary structure prediction using stochastic context-free grammars. Nucl Acids Res. 2003;31:3423–3428. 12824339 10.1093/nar/gkg614 PMC169020

[10] Michel F, Costa M, Massire C, Westhof E. Modeling RNA tertiary structure from patterns of sequence variation. Meth Enzymol. 2000;317:491–510. 10.1016/s0076-6879(00)17031-4 10829297

[11] Williams KP, Bartel DP. Phylogenetic analysis of tmRNA secondary structure. RNA. 1996;2:1306–1310. 8972778 PMC1369456

[12] Eddy SR, Durbin R. RNA sequence analysis using covariance models. Nucl Acids Res. 1994;22:2079–2088. 8029015 10.1093/nar/22.11.2079 PMC308124

[13] Gautheret D, Lambert A. Direct RNA motif definition and identification from multiple sequence alignments using secondary structure profiles. J Mol Biol. 2001;313:1003–1011. 11700055 10.1006/jmbi.2001.5102

[14] Nawrocki EP, Eddy SR. Infernal 1.1: 100-fold faster RNA homology searches. Bioinformatics. 2013;29:2933–2935. 24008419 10.1093/bioinformatics/btt509 PMC3810854

[15] Bernhart SH, Hofacker IL, Will S, Gruber AR, Stadler PF. RNAalifold: Improved consensus structure prediction for RNA alignments. BMC Bioinformatics. 2008;9:474. 19014431 10.1186/1471-2105-9-474 PMC2621365

[16] Dowell RD, Eddy SR. Efficient pairwise RNA structure prediction and alignment using sequence alignment constraints. BMC Bioinformatics. 2006;7:400. 16952317 10.1186/1471-2105-7-400 PMC1579236

[17] Parker BJ, Moltke I, Roth A, New families of human regulatory RNA structures identified by comparative analysis of vertebrate genomes. Genome Res. 2011;21:1929–1943. 21994249 10.1101/gr.112516.110 PMC3205577

[18] Rivas E, Klein RJ, Jones TA, Eddy SR. Computational identification of noncoding RNAs in *E. coli* by comparative genomics. Curr Biol. 2001;11:1369–1373. 11553332 10.1016/s0960-9822(01)00401-8

[19] Washietl S, Hofacker IL, Stadler PF. Fast and reliable prediction of noncoding RNAs. Proc Natl Acad Sci U S A. 2005;102: 2454–2459. 15665081 10.1073/pnas.0409169102 PMC548974

[20] Yao Z, Weinberg Z, Ruzzo WL. CMfinder—A covariance model based RNA motif finding algorithm. Bioinformatics. 2006;22:445–452. 16357030 10.1093/bioinformatics/btk008

[21] Deigan KE, Li TW, Mathews DH, Weeks KM. Accurate SHAPE-directed RNA structure determination. Proc Natl Acad Sci U S A. 2009;106:97–102. 19109441 10.1073/pnas.0806929106 PMC2629221

[22] Hajdin CE, Bellaousov S, Huggins W, Leonard CW, Mathews DH, Weeks KM. Accurate SHAPE-directed RNA secondary structure modeling, including pseudoknots. Proc Natl Acad Sci U S A. 2013;110:5498–5503. 23503844 10.1073/pnas.1219988110 PMC3619282

[23] Loughrey D, Watters KE, Settle AH, Lucks JB. SHAPE-Seq 2.0: Systematic optimization and extension of high-throughput chemical probing of RNA secondary structure with next generation sequencing. Nucleic Acids Res. 2014; 42(21):e165. 25303992 10.1093/nar/gku909 PMC4245970

[24] Morandi E, Manfredonia I, Simon LM, Genome-scale deconvolution of RNA structure ensembles. Nat Methods. 2021;18:249–252. 33619392 10.1038/s41592-021-01075-w

[25] Tomezsko P, Swaminathan H, Rouskin S. DMS-MaPseq for genome-wide or targeted RNA structure probing in vitro and in vivo. Methods Mol Biol. 2021;2254:219–238. 33326078 10.1007/978-1-0716-1158-6_13

[26] Vicens Q, Kieft J. Thoughts on how to think (and talk) about RNA structure. Proc Natl Acad Sci U S A. 2022;119: e2112677119. 10.1073/pnas.2112677119 PMC9169933 35439059

[27] Mauger DM, Cabral BJ, Presnyak V, mRNA structure regulates protein expression through changes in functional half-life. PNAS. 2019;116:24075–24083. 31712433 10.1073/pnas.1908052116 PMC6883848

[28] Kalvari I, Nawrocki E, Ontiveros-Palacios N, Rfam 14: Expanded coverage of metagenomic, viral, and microRNA families. NAR. 2020;gkaa1047:D192–D200. 10.1093/nar/gkaa1047 PMC7779021 33211869

[29] Dutheil JY. Detecting coevolving positions in a molecule: Why and how to account for phylogeny. Brief Bioinformatics. 2012;13:228–243. 21949241 10.1093/bib/bbr048

[30] Tillier ERM, Lui TWH. Using multiple interdependency to separate functional from phylogenetic correlations in protein alignments. Bioinformatics. 2002;19:750–755. 10.1093/bioinformatics/btg072 12691987

[31] Rivas E, Clements J, Eddy SR. A statistical test for conserved RNA structure shows lack of evidence for structure in lncRNAs. Nat Methods. 2017;14:45–48. 27819659 10.1038/nmeth.4066 PMC5554622

[32] Rivas E, Clements J, Eddy SR. Estimating the power of sequence covariation for detecting conserved RNA structure. Bioinformatics. 2020;36(02):3072–3076. 32031582 10.1093/bioinformatics/btaa080 PMC7214042

[33] Fitch WM. Toward defining the course of evolution: Minimum change for a specific tree topology. Syst Zool. 1971;20: 406–416.

[34] Rivas E RNA structure prediction using positive and negative evolutionary information. PLoS Comput Biol. 2020;16(10): e1008387. 10.1371/journal.pcbi.1008387 PMC7657543 33125376

[35] Derrien T, Johnson R, Bussotti G, The GENCODE v7 catalog of human long noncoding RNAs: Analysis of their gene structure, evolution, and expression. Genome Res. 2012; 22:1775–1789. 22955988 10.1101/gr.132159.111 PMC3431493

[36] Fang S, Zhang LL, Guo JC, NONCODEV5: A comprehensive annotation database for long non-coding RNAs. NAR. 2018;46:D308–D314. 29140524 10.1093/nar/gkx1107 PMC5753287

[37] Hon CC, Ramilowski JA, Harshbarger J, An atlas of human long non-coding RNAs with accurate 5′ ends. Nature. 2017;543:199–204. 28241135 10.1038/nature21374 PMC6857182

[38] Eddy SR. Computational analysis of conserved RNA secondary structure in transcriptomes and genomes. Annu Rev Biophys. 2014;43:433–456. 24895857 10.1146/annurev-biophys-051013-022950 PMC5541781

[39] Nojima T, Proudfoot NJ. Mechanisms of lncRNA biogenesis as revealed by nascent transcriptomics. Nat Rev Mol Cell Biol. 2022;23:389–406. 35079163 10.1038/s41580-021-00447-6

[40] Schlackow M, Nojima T, Gomes T, Dhir A, Carmo-Fonseca M, Proudfoot N. Distinctive patterns of transcription and RNA processing for human lincRNAs. Mol Cell. 2017;65: 25–38. 28017589 10.1016/j.molcel.2016.11.029 PMC5222723

[41] Johnsson P, Ziegenhain C, Hartmanis L, Transcriptional kinetics and molecular functions of long noncoding RNAs. Nat Genet. 2022;54:306–317. 35241826 10.1038/s41588-022-01014-1 PMC8920890

[42] Rivas E, Eddy SR. Secondary structure alone is generally not statistically significant for the detection of noncoding RNAs. Bioinformatics. 2000;6:583–605. 10.1093/bioinformatics/16.7.583 11038329

[43] Parker BJ, Moltke I, Roth A, . New families of human regulatory RNA structures identified by comparative analysis of vertebrate genome. Genome Res. 2011;21:1929–1943. 21994249 10.1101/gr.112516.110 PMC3205577

[44] Seemann SE, Mirza AH, Hansen C, The identification and functional annotation of RNA structures conserved in vertebrates. BMC Genomics. 2017;27:1371–1383. 10.1101/gr.208652.116 PMC5538553 28487280

[45] Washietl S, Hofacker IL, Lukasser M, Hüttenhofer A, Stadler PF. Mapping of conserved RNA secondary structures predicts thousands of functional noncoding RNAs in the human genome. Nat Biotechnol. 2005;23:1383–1390. 16273071 10.1038/nbt1144

[46] Washietl S, Pedersen JS, Korbel JO, Structured RNAs in the ENCODE selected regions of the human genome. Genome Res. 2007;17:852–864. 17568003 10.1101/gr.5650707 PMC1891344

[47] Gao W, Jones TA, Rivas E. Discovery of 17 novel conserved structural RNAs in fungi. NAR. 2021;49:6128–6143. 34086938 10.1093/nar/gkab355 PMC8216456

[48] Moler C, Van Loan C. Nineteen dubious ways to compute the exponential of a matrix. SIAM Rev. 1978;20(4):801–836.

[49] Andronescu M, Aguirre-Hernandez R, Condon A, Hoos HH. RNAsoft: A suite of RNA secondary structure prediction and design software tools. NAR. 2003;31:3416–3422. 12824338 10.1093/nar/gkg612 PMC169018

[50] Ding Y, Chan CY, Lawrence CE. Sfold web server for statistical folding and rational design of nucleic acids. Nucleic Acids Res. 2004;32:W135–W141. 15215366 10.1093/nar/gkh449 PMC441587

[51] Dirks RM, Pierce NA. A partition function algorithm for nucleic acid secondary structure including pseudoknots. J Comput Chem. 2003;24:1664–1177. 12926009 10.1002/jcc.10296

[52] Do CB, Woods DA, Batzoglou S. CONTRAfold: RNA secondary structure prediction without physics-based models. Bioinformatics. 2006;22:e90–e98. 16873527 10.1093/bioinformatics/btl246

[53] Dowell RD, Eddy SR. Evaluation of several lightweight stochastic context-free grammars for RNA secondary structure prediction. BMC Bioinformatics. 2004;5:71. 15180907 10.1186/1471-2105-5-71 PMC442121

[54] Gruber AR, Bernhart SH, Lorenz R. The ViennaRNA web services. Methods Mol Biol. 2015;1269:307–26. 10.1007/978-1-4939-2291-8_19 25577387

[55] Reuter JS, Mathews DH. RNAstructure: Software for RNA secondary structure prediction and analysis. BMC Bioinformatics. 2010;11:10. 20230624 10.1186/1471-2105-11-129 PMC2984261

[56] Rivas E, Lang R, Eddy SR. A range of complex probabilistic models for RNA secondary structure prediction that include the nearest neighbor model and more. RNA. 2012;18:193–212. 22194308 10.1261/rna.030049.111 PMC3264907

[57] Swenson MS, Anderson J, Ash A, GTfold: Enabling parallel RNA secondary structure prediction on multi-core desktops. BMC Res Notes. 2012;5:341. 22747589 10.1186/1756-0500-5-341 PMC3748833

[58] Zuker M On finding all suboptimal foldings of an RNA molecule. Science. 1989;244:48–52. 2468181 10.1126/science.2468181

[59] Spitale RC, Crisalli P, Flynn RA, Torre EA, Kool ET, Chang HY. RNA SHAPE analysis in living cells. Nat Chem Biol. 2013;9:18–20. 23178934 10.1038/nchembio.1131 PMC3706714

[60] Rouskin S, Zubradt M, Washietl S, Kellis M, Weissman JS. Genome-wide probing of RNA structure reveals active unfolding of mRNA structures in vivo. Nature. 2014;505:701–705. 24336214 10.1038/nature12894 PMC3966492

[61] Rangan R, Zheludev IN, Das R. RNA genome conservation and secondary structure in SARS-CoV-2 and SARS-related viruses: A first look. RNA. 2020;26:937–959. 32398273 10.1261/rna.076141.120 PMC7373990

[62] Manfredonia I, Nithin C, Ponce-Salvatierra A, Genomewide mapping of SARS-CoV-2 RNA structures identifies therapeutically-relevant elements. Nucl Acids Res. 2020;48: 12436–12452. 33166999 10.1093/nar/gkaa1053 PMC7736786

[63] Shannon CE. A note on the concept of entropy. Bell System Tech J. 1948;27:379–423.

[64] Woolf B The log likelihood ratio test (the G-test). Ann Hum Genet. 1957;21:397–409. 13435648 10.1111/j.1469-1809.1972.tb00293.x

[65] Dunn SD, Wahl LM, Gloor GB. Mutual information without the influence of phylogeny or entropy dramatically improves residue contact predictions. Bioinformatics. 2007;24:333–340. 18057019 10.1093/bioinformatics/btm604

[66] Lindgreen S, Gardner PP, Krogh A. Measuring covariation in RNA alignments: Physical realism improves information measures. Bioinformatics. 2006;22:2988–2995. 17038338 10.1093/bioinformatics/btl514

[67] Fields DS, Gutell RR. An analysis of large rRNA sequences folded by a thermodynamic method. Fold Des. 1996;1:419–430. 9080188 10.1016/S1359-0278(96)00058-2

[68] Gutell RR, Larsen N, Woese CR. Lessons from an evolving rRNA: 16 S and 23 S rRNA structures from a comparative perspective. Microbiol Rev. 1994;58:10–26. 8177168 10.1128/mr.58.1.10-26.1994 PMC372950

[69] Hofacker IL, Fekete M, Stadler PF. Secondary structure prediction for aligned RNA sequences. J Mol Biol. 2002;319: 1059–1066. 12079347 10.1016/S0022-2836(02)00308-X

[70] Rivas E, Eddy SR. Response to Tavares et al., “Covariation analysis with improved parameters reveals conservation in lncRNA structures”. bioRxiv, 2018. 10.1101/2020.02.18.955047. PMC6515926 30890332

[71] Tavares RCA, Pyle AM, Somarowthu S. Phylogenetic analysis with improved parameters reveals conservation in lncRNA structures. J Mol Biol. 2019;431(8):1592–1603. 30890332 10.1016/j.jmb.2019.03.012 PMC6515926

[72] Uroda T, Anastasakou E, Rossi A, Conserved pseudoknots in lncRNA MEG3 are essential for stimulation of the p53 pathway. Mol Cell. 2019;75:982–995. 31444106 10.1016/j.molcel.2019.07.025 PMC6739425

[73] Weigt M, White RA, Szurmant H, Hoch JA, Hwa T. Identification of direct residue contacts in protein–protein interaction by message passing. Proc Natl Acad Sci U S A. 2009;106:67–72. 19116270 10.1073/pnas.0805923106 PMC2629192

[74] Ekeberg M, Lövkvist C, Lan Y, Weigt M, Aurell E. Improved contact prediction in proteins: Using pseudolikelihoods to infer Potts models. Phys Rev E. 2013;87(1):012707. 10.1103/PhysRevE.87.012707 23410359

[75] Jones DT, Buchan DWA, Cozzetto D, Pontil M. PSICOV: Precise structural contact prediction using sparse inverse covariance estimation on large multiple sequence alignments. Bioinformatics. 2012;28(2):184–190. 22101153 10.1093/bioinformatics/btr638

[76] Kamisetty H, Ovchinnikov S, Baker D. Assessing the utility of coevolution-based residue–residue contact predictions in a sequence-and structure-rich era. Proc Natl Acad Sci. 2013; 110(39):15674–15679. 24009338 10.1073/pnas.1314045110 PMC3785744

[77] Marks DS, Colwell LJ, Sheridan R, Protein 3D structure computed from evolutionary sequence variation. PLoS One. 2011;6(12):e28766. 22163331 10.1371/journal.pone.0028766 PMC3233603

[78] Morcos F, Pagnani A, Lunt B, Direct-coupling analysis of residue coevolution captures native contacts across many protein families. Proc Natl Acad Sci U S A. 2011;108:E1293–E1301. 22106262 10.1073/pnas.1111471108 PMC3241805

[79] Cuturello F, Tiana G, Bussi G. Assessing the accuracy of direct-coupling analysis for RNA contact prediction. RNA. 2020;26:637–647. 32115426 10.1261/rna.074179.119 PMC7161351

[80] De Leonardis E, Lutz B, Ratz S, Direct-coupling analysis of nucleotide coevolution facilitates RNA secondary and tertiary structure prediction. Nucl Acids Res. 2015;43:10444–10455. 26420827 10.1093/nar/gkv932 PMC4666395

[81] Weinreb C, Riesselman AJ, Ingraham JB, Gross T, Sander C, Marks DS. 3D RNA and functional interactions from evolutionary couplings. Cell. 2016;165:963–975. 27087444 10.1016/j.cell.2016.03.030 PMC5024353

[82] Rivas E Evolutionary conservation of RNA sequence and structure. WIREs RNA. 2021;12:e1649. 33754485 10.1002/wrna.1649 PMC8250186

[83] Vaswani A, Shazeer N, Parmar N, . Attention is all you need. arXiv preprint arXiv:1706.03762. 2017.

[84] Jumper J, Evans R, Pritzel A, Highly accurate protein structure prediction with AlphaFold. Nature. 2021;596: 583–589. 34265844 10.1038/s41586-021-03819-2 PMC8371605

[85] Pearce R, Omenn GS, Zhang Y. De novo RNA tertiary structure prediction at atomic resolution using geometric potentials from deep learning. bioRxiv, page 2022.05.15.491755, 2022.

[86] Miao Z, Adamiak RW, Antczak M, RNA-puzzles round III: 3D RNA structure prediction of five riboswitches and one ribozyme. RNA. 2017;23:655–672. 28138060 10.1261/rna.060368.116 PMC5393176

[87] Miao Z, Adamiak RW, Antczak M, RNA-puzzles round IV: 3D structure prediction of four ribozymes and two aptamers. RNA. 2020;26:982–995. 32371455 10.1261/rna.075341.120 PMC7373991

[88] Jones AN, Pisignano G, Pavelitz T, An evolutionarily-conserved RNA structure in the functional core of the lincRNA Cyrano. RNA. 2020;26:1234–1246. 10.1261/rna.076117.120. 32457084 PMC7430676

[89] Seemann SE, Mirza AH, Bang-Berthelsen CH, Does rapid sequence divergence preclude RNA structure conservation in vertebrates? NAR. 2022;50:2452–2463. 35188540 10.1093/nar/gkac067 PMC8934657

[90] Haeussler M, Zweig AS, Tyner C, The UCSC genome browser database: 2019 update. Nucl Acids Res. 2019;47(D1): D853–D858. 30407534 10.1093/nar/gky1095 PMC6323953

[91] Gao W. Discovery and functional characterization of noncoding RNAs in eukaryotic genomes. B.S. Thesis. Cambridge, USA: Harvard University, 2020.

[92] Cooper HB, Gardner PP. Features of functional human genes. bioRxiv, 2020.

[93] Wheeler TJ, Eddy SR. Nhmmer: DNA homology search with profile HMMs. Bioinformatics. 2013;29:2487–2489. 23842809 10.1093/bioinformatics/btt403 PMC3777106

[94] International Human Genome Sequencing Consortium. Initial sequencing and analysis of the human genome. Nature. 2001;409:860–921. 11237011 10.1038/35057062

[95] Jacq C, Miller JR, Brownlee GG. A pseudogene structure in 5 S DNA of *Xenopus laevis*. Cell. 1977;12:109–120. 561661 10.1016/0092-8674(77)90189-1

[96] Hawkes EJ, Hennelly SP, Novikova IV, Irwin JA, Dean C, Sanbonmatsu KY. COOLAIR antisense RNAs form evolutionarily conserved elaborate secondary structures. Cell Rep. 2016;16:3087–3096. 27653675 10.1016/j.celrep.2016.08.045 PMC6827332

[97] Karlin S, Altschul SF. Methods for assessing the statistical significance of molecular sequence features by using general scoring schemes. Proc Natl Acad Sci U S A. 1990;87: 2264–2268. 2315319 10.1073/pnas.87.6.2264 PMC53667

[98] Gray NK, Pantopoulos K, Dandekar T, Ackrell BA, Hentze MW. Translational regulation of mammalian and drosophila citric acid cycle enzymes via iron-responsive elements. PNAS. 1996;93:4925–4930. 8643505 10.1073/pnas.93.10.4925 PMC39381

[99] Walczak R, Westhof E, Carbon P, Krol A. A novel RNA structural motif in the selenocysteine insertion element of eukaryotic selenoprotein mRNAs. RNA. 1996;2:367–337. 8634917 PMC1369379

[100] Yang A Discovery of conserved structural noncoding RNAs in pufferfish. B.S. thesis. Cambridge, USA: Harvard University, 2022.

[101] Li S, Breaker RR. Identification of 15 candidate structured noncoding RNA motifs in fungi by comparative genomics. BMC Genomics. 2017;18(1):785. 29029611 10.1186/s12864-017-4171-y PMC5640933

[102] Somarowthu S, Legiewicz M, Chillón I, Marcia M, Liu F, Pyle AM. HOTAIR forms an intricate and modular secondary structure. Mol Cell. 2015;58:353–361. 25866246 10.1016/j.molcel.2015.03.006 PMC4406478

[103] Moler C, Van Loan C. Nineteen dubious ways to compute the exponential of a matrix, twenty-five years later. SIAM Rev. 2003;45:3–49.

