# Supplementary material for: Thirteen dubious ways to detect conserved structural RNAs
Source: IUBMB Life. Author manuscript; Available in PMC 2024 Jul 10. (PMC11234323; doi:10.1002/iub.2694)

RF00001.invertebrates.Eval_1e-10_1.select896.cacofold

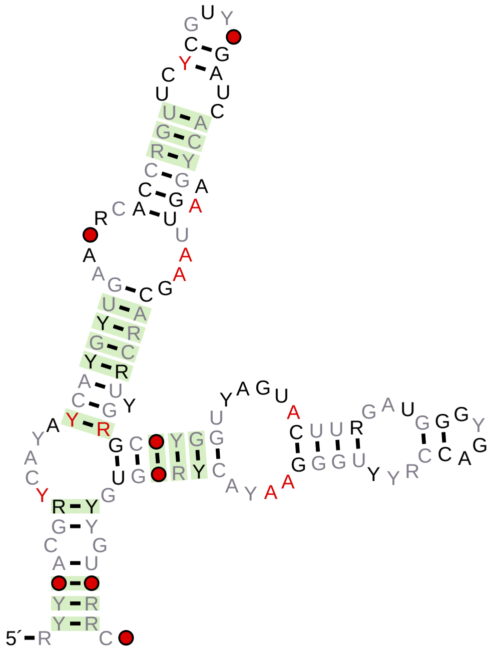

Supplement: supplementary_material [file NIHMS1907110-supplement-supplementary_material.gz › supplemental_material/Figure7/RF00001.invertebrates.Eval_1e-10_1.select896.cacofold.R2R.sto.pdf]
