# Supplementary figures and images for: Thirteen dubious ways to detect conserved structural RNAs
Source: IUBMB Life. Author manuscript; Available in PMC 2024 Jul 10. (PMC11234323; doi:10.1002/iub.2694)

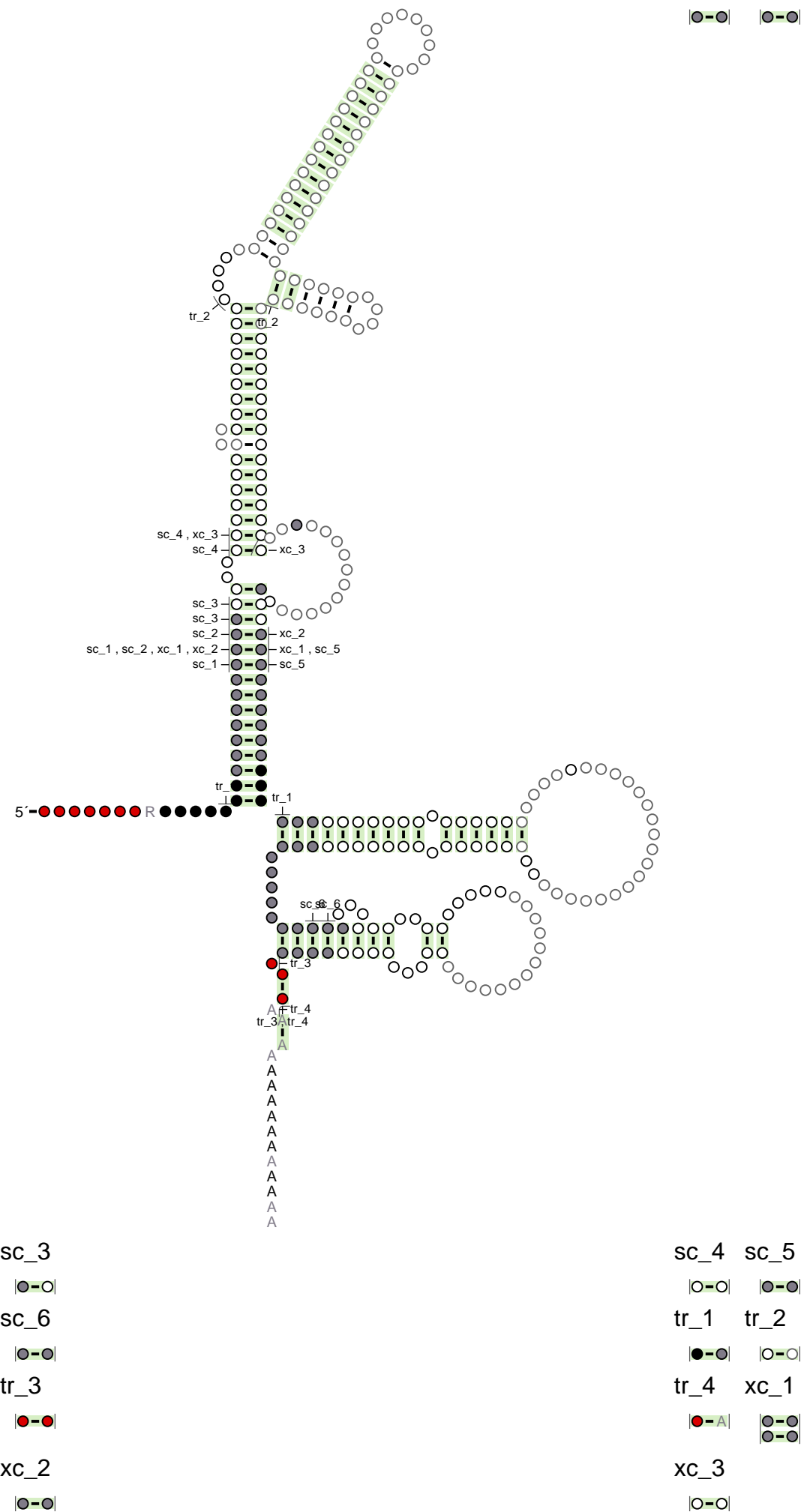

Supplement: supplementary_material [file NIHMS1907110-supplement-supplementary_material.gz › supplemental_material/Figure9/R-scape/CDKN1B-3UTR_human_fig1_invertebrates.Eval_1.0_1.fold.R2R.sto.pdf]

# Fig1\_toy\_caco\_1.cacofold

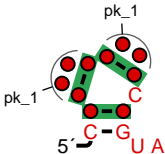

pk\_1

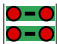

Supplement: supplementary_material [file NIHMS1907110-supplement-supplementary_material.gz › supplemental_material/Figure1/R-scape/Fig1_toy_caco_1.cacofold.R2R.sto.pdf]

# HOTAIR\_H11\_1.cacofold

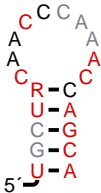

Supplement: supplementary_material [file NIHMS1907110-supplement-supplementary_material.gz › supplemental_material/Figure4/HOTAIR/R-scape/HOTAIR_H11_1.cacofold.R2R.sto.pdf]

# HOTAIR\_H11\_1

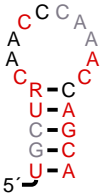

Supplement: supplementary_material [file NIHMS1907110-supplement-supplementary_material.gz › supplemental_material/Figure4/HOTAIR/R-scape/HOTAIR_H11_1.R2R.sto.pdf]

HOTAIR\_D1\_1

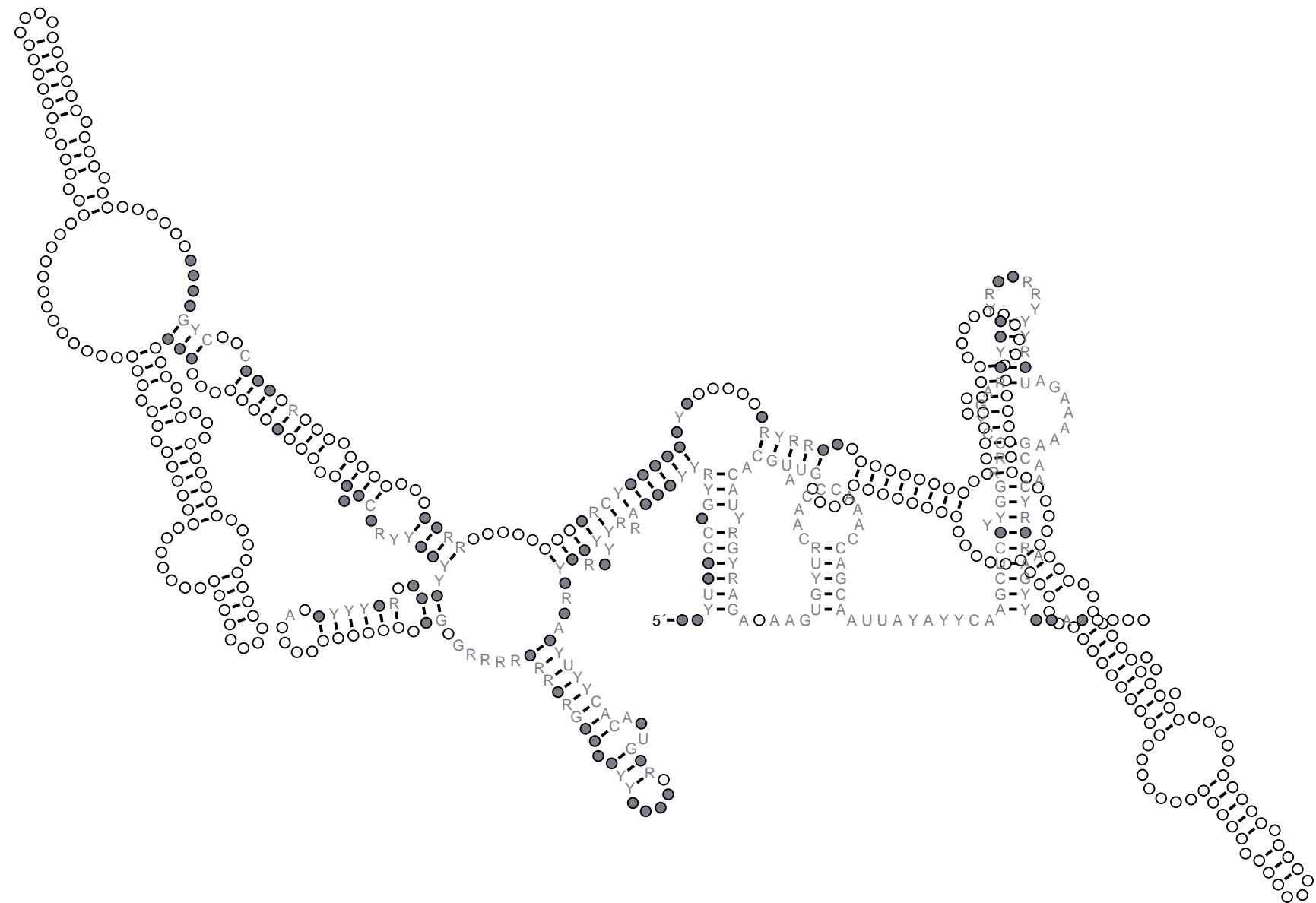

Supplement: supplementary_material [file NIHMS1907110-supplement-supplementary_material.gz › supplemental_material/Figure4/HOTAIR/R-scape/HOTAIR_D1_1.R2R.sto.pdf]

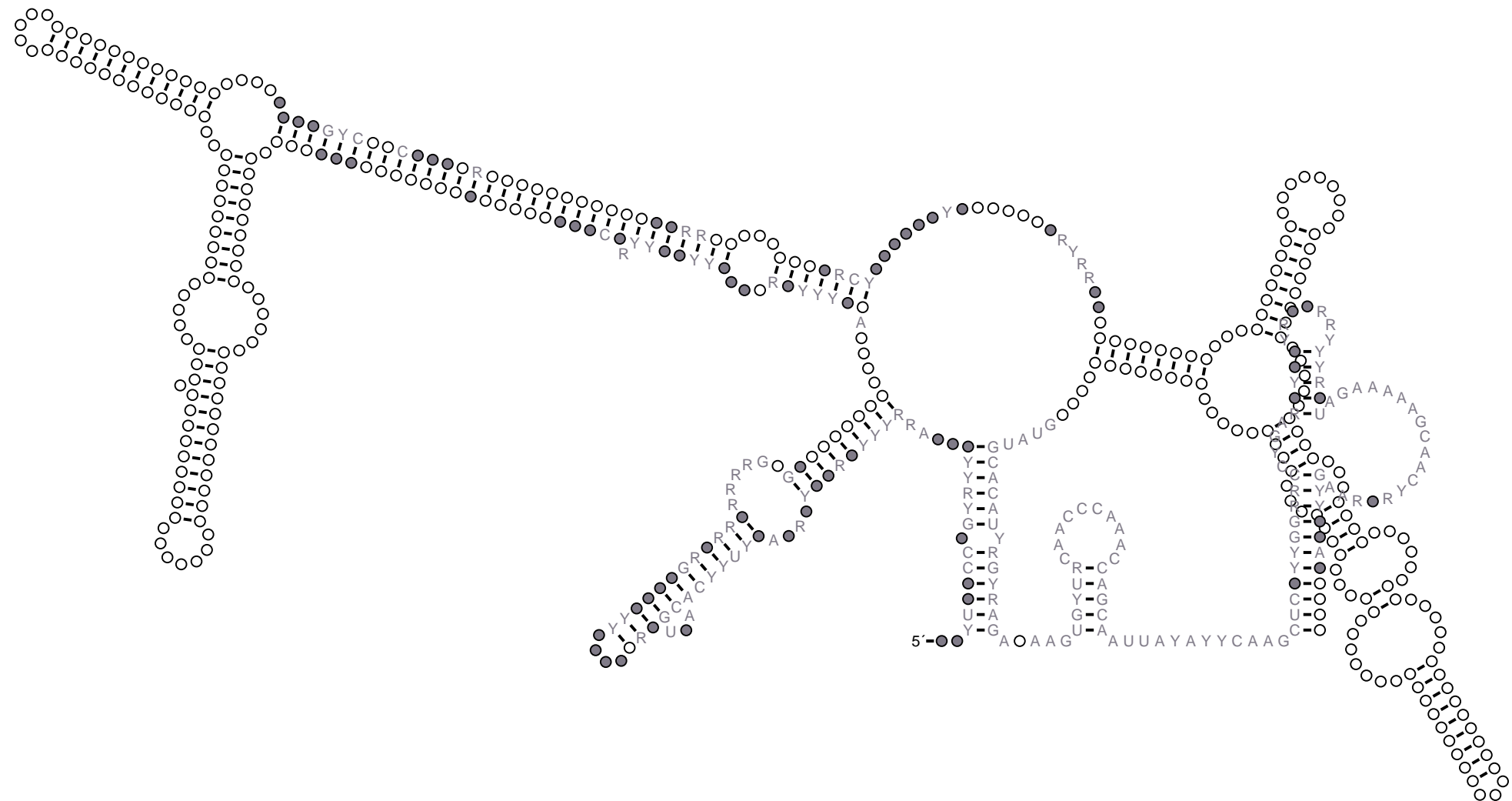

Supplement: supplementary_material [file NIHMS1907110-supplement-supplementary_material.gz › supplemental_material/Figure4/HOTAIR/R-scape/HOTAIR_D1_1.cacofold.R2R.sto.pdf]

# E3-Infernal\_41sequences.H11.273-298\_1

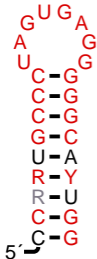

Supplement: supplementary_material [file NIHMS1907110-supplement-supplementary_material.gz › supplemental_material/Figure4/MEG3/R-scape/E3-Infernal_41sequences.H11.273-298_1.R2R.sto.pdf]

E3-Infernal\_41sequences\_1.cacofold

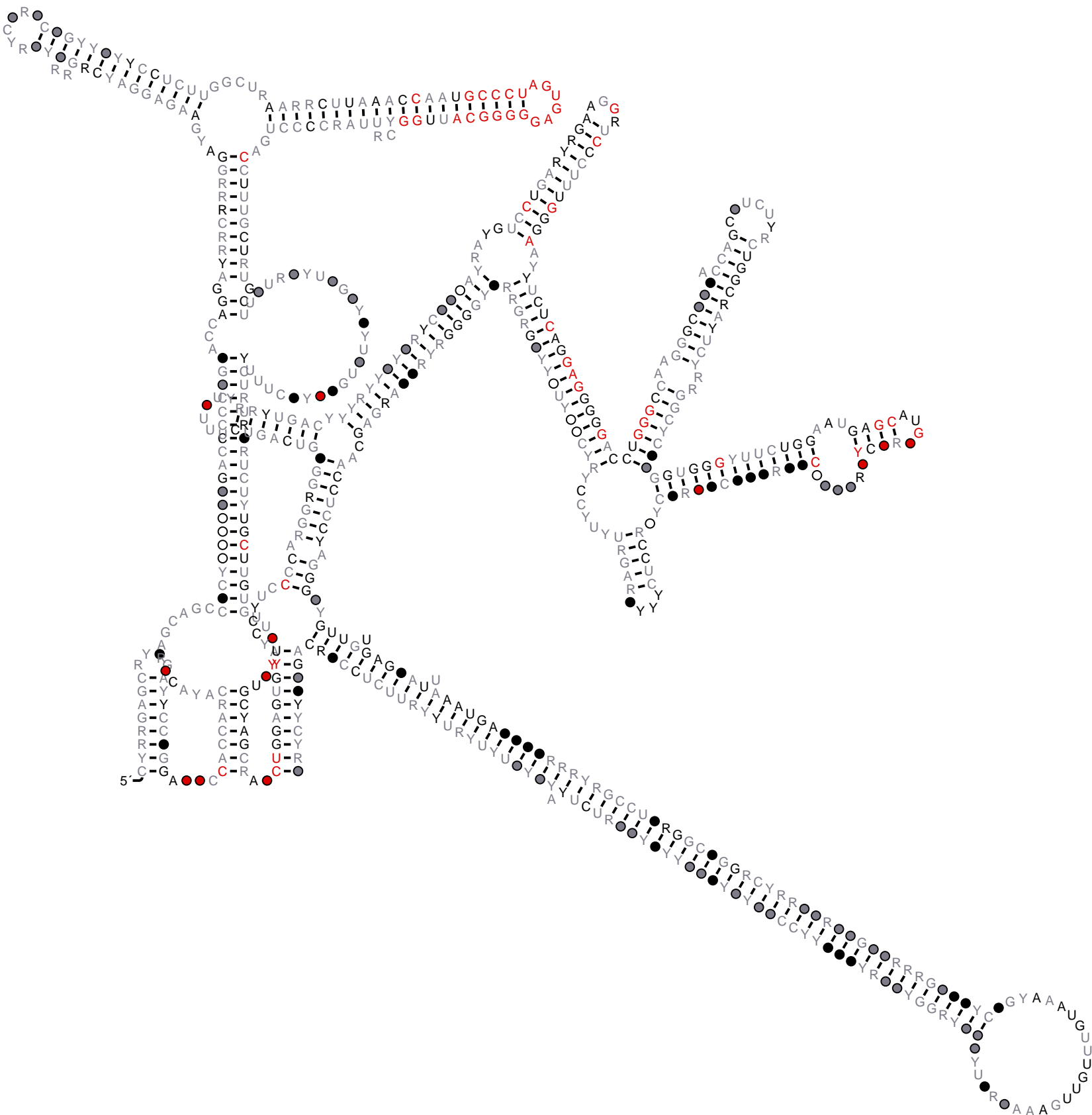

Supplement: supplementary_material [file NIHMS1907110-supplement-supplementary_material.gz › supplemental_material/Figure4/MEG3/R-scape/E3-Infernal_41sequences_1.cacofold.R2R.sto.pdf]

E3-Infernal\_41sequences.H11.273-298\_1.cacofold

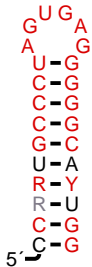

Supplement: supplementary_material [file NIHMS1907110-supplement-supplementary_material.gz › supplemental_material/Figure4/MEG3/R-scape/E3-Infernal_41sequences.H11.273-298_1.cacofold.R2R.sto.pdf]

## E3-Infernal\_41sequences\_1

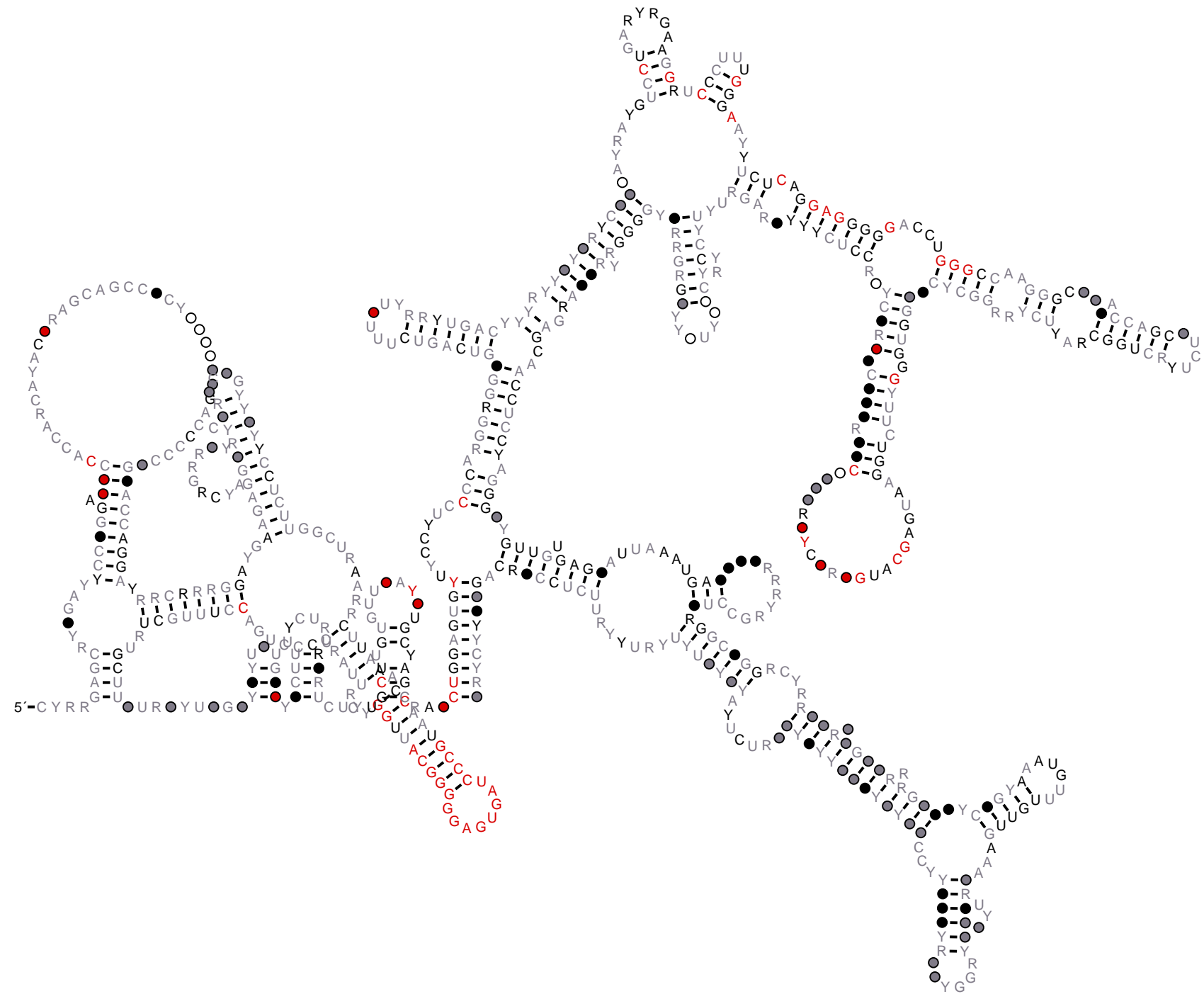

Supplement: supplementary_material [file NIHMS1907110-supplement-supplementary_material.gz › supplemental_material/Figure4/MEG3/R-scape/E3-Infernal_41sequences_1.R2R.sto.pdf]

# Fig1\_toy\_caco\_1.cacofold

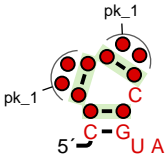

pk\_1

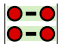

Supplement: supplementary_material [file NIHMS1907110-supplement-supplementary_material.gz › supplemental_material/Figure2/R-scape/Fig1_toy_caco_1.cacofold.R2R.sto.pdf]
