# Supplementary material for: Thirteen dubious ways to detect conserved structural RNAs
Source: IUBMB Life. Author manuscript; Available in PMC 2024 Jul 10. (PMC11234323; doi:10.1002/iub.2694)

CDKN1B-3UTR_human_fig1_invertebrates.Eval_1.0_1

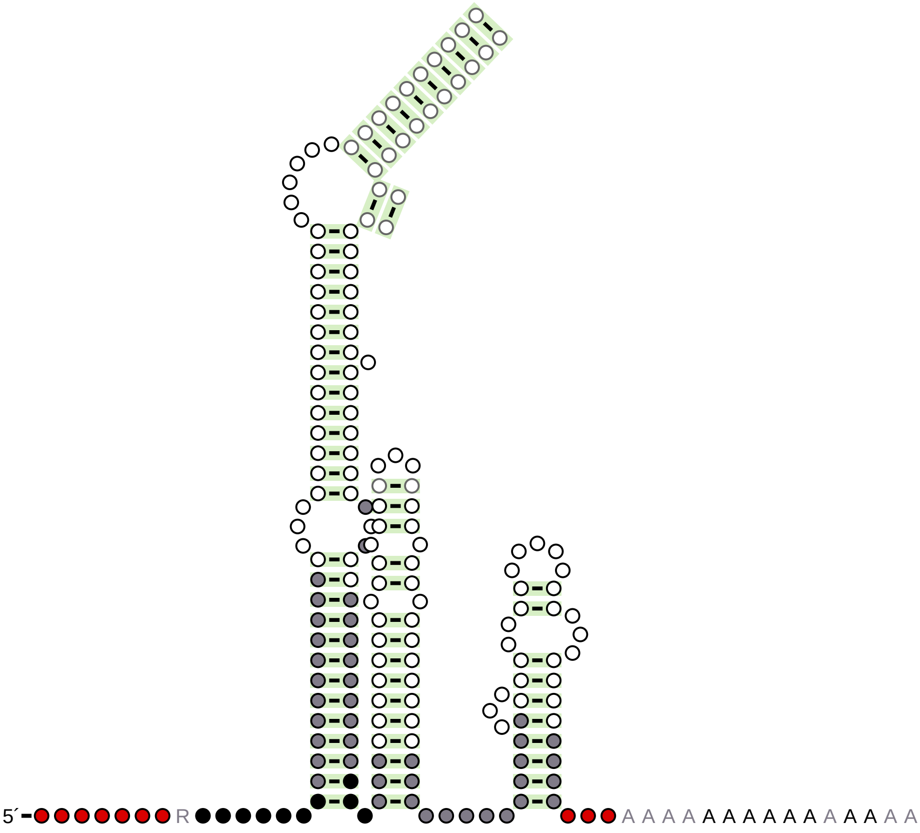

Supplement: supplementary_material [file CDKN1B-3UTR_human_fig1_invertebrates.Eval_1.0_1.R2R.sto.pdf › supplemental_material/Figure9/R-scape/CDKN1B-3UTR_human_fig1_invertebrates.Eval_1.0_1.R2R.sto.pdf]
